# Effects of Feeding Milk Replacer *Ad Libitum* or in Restricted Amounts for the First Five Weeks of Life on the Growth, Metabolic Adaptation, and Immune Status of Newborn Calves

**DOI:** 10.1371/journal.pone.0168974

**Published:** 2016-12-30

**Authors:** Christine T. Schäff, Jeannine Gruse, Josefine Maciej, Manfred Mielenz, Elisa Wirthgen, Andreas Hoeflich, Marion Schmicke, Ralf Pfuhl, Paulina Jawor, Tadeusz Stefaniak, Harald M. Hammon

**Affiliations:** 1 Institute of Nutritional Physiology “Oskar Kellner”, Leibniz Institute for Farm Animal Biology (FBN), Dummerstorf, Germany; 2 Institute for Genome Biology, Leibniz Institute for Farm Animal Biology (FBN), Dummerstorf, Germany; 3 Ligandis GbR, Gülzow-Prüzen, Germany; 4 Clinic for Cattle, University of Veterinary Medicine, Foundation, Hannover, Germany; 5 Institute for Muscle Biology and Growth, Leibniz Institute for Farm Animal Biology (FBN), Dummerstorf, Germany; 6 Department of Immunology, Pathophysiology and Veterinary Preventive Medicine, Faculty of Veterinary Medicine, Wroclaw University of Environmental and Life Sciences, Wroclaw, Poland; University of British Columbia, CANADA

## Abstract

The pre-weaning period is critical for calf health and growth, and intensive milk feeding programs may assist postnatal development by improving body growth and organ maturation. The aim of the present work was to study the effects of *ad libitum* milk replacer (MR) feeding on the growth, metabolic adaptation, health, and immune status of newborn calves. Twenty-eight newborn Holstein and Holstein x Charolais crossbred calves were fed *ad libitum* (ADLIB) or in restricted amounts (6 liters per day; RES) during the first five weeks of life. The MR intake in the ADLIB treatment was gradually reduced at weeks 6 and 7, and all calves then received 6 liters of MR per day until day 60. Blood samples were collected to measure the plasma concentrations of metabolites, insulin, insulin-like growth factor (IGF)-I and IGF binding proteins (IGFBP), immunoglobulins, and acute phase proteins. The expression of mRNA associated with both the somatotropic axis and gluconeogenic enzymes was measured in the liver on day 60. Intensive feeding improved MR intake and growth in ADLIB without influencing concentrate intake. Carcass weight, perirenal fat, and muscle mass were greater in ADLIB. Plasma concentrations of glucose, triglycerides, insulin, and IGF-I were greater, whereas plasma concentrations of β-hydroxybutyrate, total protein, albumin, urea, IGFBP-2 and -4, and fibrinogen were lower at distinct time points in ADLIB. The hepatic mRNA expression of cytosolic phosphoenolpyruvate carboxykinase was greater in ADLIB. Most metabolic and endocrine differences occurred during the MR feeding period, but a slightly greater concentrate intake was associated with increased plasma IGF-I and insulin at the end of the study. The immune and health status of the calves were not affected by MR feeding. However, increased plasma fibrinogen in the RES group suggested differences in the acute phase response.

## Introduction

The rearing of the pre-weaning calf is one of the most critical issues in cattle breeding, and calf losses during the neonatal period remain high [1–4]. Recent discussions have focused on the milk or milk replacer (MR) feeding regimen in newborn calves to stimulate postnatal growth and development through intensive nutrient intake to improve organ development, structural growth, health, and well-being [5–9]. Intensive milk or MR feeding programs resulted in an elevated dry matter and energy intake and body growth during the pre-weaning period [5,7,10–13]. It has been suggested that calves with elevated milk or MR intake during the pre-weaning period are less susceptible to illness [14,15]. Although intensive milk feeding regimen increases growth rates compared with a feeding regimen with restricted milk intake [10–13,16], there are concerns about such intensive milk feeding programs regarding the effects of low solid feed intake and impaired rumen development when calves are fed high amounts of milk [11,17–19]. Thus, sufficient concentrate intake pre-weaning is necessary to maintain constant growth and weight gain after weaning [20,21], and too much milk intake might promote loose feces and diarrhea [16]. However, too many restrictions on milk feeding leads to impaired growth, higher losses of calves, and behavioral aberrances [9,10,22]. In addition, intensive milk feeding and accelerated growth in pre-weaned calves affect subsequent milk performance, indicating a long-lasting impact of the pre-weaning growth period on the life-time performance of dairy cows [20,23]. Therefore, there is great interest in understanding the consequences of intensive milk or MR feeding on the pre-weaning growth, development, and health of calves.

The feeding management of calves, starting with the colostral period, influences the maturation of the postnatal somatotropic axis [24–26]. The growth hormone (GH)—insulin-like growth factor (IGF) axis is an important regulator of postnatal growth and development in cattle, including the development of the mammary gland [27–29]. In addition, the GH-IGF axis stimulates immune function [30], which could affect the postnatal immune response and health of calves. Previous studies on the development of the somatotropic axis with respect to different milk-feeding protocols indicated an elevated plasma IGF-I concentration in calves [5,7,13,31–33]. Because the somatotropic axis depends on the nutrient intake [34], the increased protein and energy intake from elevated milk or MR feeding stimulates IGF-I secretion and somatotropic axis maturation [35–37]. The plasma concentrations of IGF-binding proteins (IGFBPs) might also be affected, as these proteins represent established biomarkers of GH action and regulate the IGF-I effects on cell proliferation and tissue growth [38]. Therefore, calves might benefit from a stimulated somatotropic axis as a result of intensive MR feeding, showing improved growth and immune status. However, it is unclear whether the stimulation of the somatotropic axis has long-term effects on growth after the cessation of intensive MR feeding and whether this effect is associated with the health and immune status of the calves.

The aim of the present study was to investigate the growth development, the metabolic status, endocrine growth regulation, and the health and immune status of pre-weaned calves fed different amounts of MR for the first five weeks of life. We hypothesized that elevated MR feeding affects the performance, metabolic traits, and the systemic and hepatic IGF system of the calves and expect benefits on the immune system.

## Materials and Methods

### Animals and Feeding

The experimental procedures were conducted according to the German Animal Welfare law, the regulation on the protection of animals used for scientific purposes (Tierschutz-Versuchstierverordnung), and the animal care guidelines of the State Government in Mecklenburg-Western Pommerania, Germany. This study was approved by the State Office for Agriculture, Food Safety, and Fisheries, Mecklenburg-Western Pommerania (LALLF; M-V/TSD/7221.3–2.1–011/12) and was performed from May to October 2012.

To compare the changes associated with different feeding practices, 28 calves (5 Holstein and 23 Holstein x Charolais crossbred calves; 19 male and 9 female) were examined and randomly divided into two different experimental groups (n = 14 per group). The calves were spontaneously born and fed colostrum for the first 3 days of life. From day 4 on, the calves received reconstituted MR (SalvaLac MiraPro 45; Salvana Tiernahrung GmbH, Klein-Offenseth Sparrieshoop, Germany; 125 g powder per liter; composition shown in Table 1). MR instead of whole milk was chosen to make sure that all calves received the same quality of milk during the whole experimental period, as we have focused on the differences in nutrient intake in this study. Therefore, a high-quality MR with protein originated only from milk protein was used.

**Table 1:** Composition of Milk Replacer (MR) and the Concentrate.

Chemical Composition	MR SalvaLac MiraPro 45^1^	Concentrate Kälber Start 18/3 pelletiert^2^
	% dry matter (DM)	
Crude protein	20	16
Crude fat	19	3.0
Lactose	51	-
Crude fiber	0.1	9.8
Metabolizable energy^3^	18.6	10.4
Crude ash	7.5	6.5
Calcium	0.8	1.0
Phosphorus	0.75	0.5
Sodium	0.55	0.2
Lysine	1.7	-

^1^ Ingredients of MR: 45% skimmed milk powder; 35% whey powder; 18% plant oil (palm and coconut oil, refined and homogenized, soybean oil); Pre-mix (additives per kg: 50,000 I.U. vitamin A; 4,000 I.U. vitamin D3; 200 mg vitamin E as α-tocopherol acetate; 120 mg iron as glycine iron chelate hydrate; 48 mg zinc as zinc oxide; 46 mg manganese as manganese (II) oxide; 9 mg copper as glycine copper chelate hydrate; 0.18 mg cobalt as cobalt (II) carbonate monohydrate; 0.5 mg iodine as water free calcium iodate; 0.4 mg selenite as sodium selenite; 1.2*10^9^ colony-forming units *Enterococcus faecium* (NCIMB 10415); 36 mg butylated hydroxytoluene (BHT); 1,800 mg polyethylene glycol (PEG) soy oil fatty acid ester).

^2^ Ingredients of concentrate: wheat gluten feed; triticale; rapeseed extraction meal; beet pulp; sunflower extraction meal from partially peeled seed; wheat bran; wheat; peeled oat bran; soybean hulls; beet molasses; linseed; soybean extraction meal; calcium carbonate; sodium chloride. Nutritional additives per kg wet weight: 10,800 I.E. vitamin A; 1,215 I.E. vitamin D as vitamin D_3_; 40 mg vitamin E; 0.7 mg iodine as water free calcium iodate; 0.18 mg cobalt as cobalt (II) carbonate monohydrate; 46 mg manganese as manganese (II) oxide; 48 mg zinc as zinc oxide; 0.4 mg selenite as sodium selenite.

^3^ Metabolizable energy is given as MJ/kg dry matter.

The calves of group ADLIB (3 Holstein and 11 Holstein x Charolais; 8 male and 6 female) received MR *ad libitum* for the first five weeks of life using an automated feeder (Förster-Technik GmbH, Engen, Germany). After weeks 5, the available amount of MR was reduced proportionally day by day to 6 liters per day at week 7 and was maintained constant thereafter. The individual reduction of MR intake was calculated by the feeding program of the automate and corresponded to ca. 71 g milk powder or 0.57 liter of MR per day. The calves of group RES (2 Holstein and 12 Holstein x Charolais; 11 male and 3 female) with restricted MR feeding received up to 6 liters of MR per day for the entire experimental period also using an automated feeder. All calves were provided free access to water and concentrate (Kälber Start 18/3; Vollkraft Mischfutterwerke GmbH, Karstädt, Germany; composition shown in Table 1; delivered by an automated feeder; Förster-Technik GmbH, Engen, Germany). Individual intake of MR and concentrates and daily amounts of MR and concentrate intake were provided by the computer program of the feeding station. MR was prepared in portions of 0.5 liters and calves were allowed to drink 2 liters per visit. Refused MR was discarded to avoid uncontrolled MR intake of calves with no right. Concentrates were offered in portions of 60 g and calves could take several portions per visit, but it was not possible to discard refusals. Total dry matter intake and intake of metabolizable energy (ME) were calculated according to Drackley [21]. To prevent anemia, all calves received 1 g of iron dextran (Ursoferran 150 mg/mL; 7 mL per os; Serumwerk Bernburg AG, Bernburg, Germany) during the first colostrum feeding. After evening feeding, the calves were fed halofuginone (Halocur; Intervet Productions S.A., Igoville, France) for 7 consecutive days (2 mL/10 kg) to prevent diarrhea resulting from *Cryptosporidium spp*. Bouts of diarrhea (scours) were treated using oral rehydration solution (Glycostar classic; WDT eG, Garbsen, Germany). The treatment days per calf were recorded and are shown in the results section and S1 Table.

### Growth, Body Composition and Tissue Sampling

The calves were weighed immediately after birth and then weekly thereafter. Data feed efficiency, namely body weight gain/dry matter intake, body weight gain/ME intake, and body weight gain/protein intake were calculated as sum of the entire experimental period, respectively. The calves were harvested prior to weaning on day 60 ± 2 through exsanguination after capital bolt stunning in the Research Institute’s abattoir. Both carcass weight and carcass composition were determined after cutting and boning. Cuts referred to the German cutting schema and are illustrated in S1 Fig [39]. The weight of the cuts, liver, pancreas, and perirenal fat, was determined. Samples of liver tissue were collected to determine the glycogen and glucose concentrations and for mRNA expression studies. The tissue was stored at -80°C until further analysis.

### Metabolites and Hormones

Blood samples were obtained through jugular vein puncture before first feeding, at 24 h after the first feeding, on the 8th day of life and weekly thereafter for up to 8 weeks. Blood sampling were taken in the morning (7.00 am) before start of the daily feeding program. The samples were collected in tubes (Vacuette; Greiner Bio-One GmbH, Kremsmünster, Austria) containing: 1.2–2 mg/mL K_3_ ethylenediaminetetraacetic acid (EDTA) to determine the plasma concentrations of insulin, IGF-I, and IGFBPs; 2–4 mg/mL sodium fluoride and 1.2–2 mg/mL K_3_ EDTA to determine the plasma concentrations of glucose, lactate, beta-hydroxybutyrate (BHB), non-esterified fatty acids (NEFA), triglycerides, cholesterol, total protein, albumin, and urea; and 12–30 IU lithium heparin to determine the plasma concentrations of fibrinogen, haptoglobin, and immunoglobulin (Ig) G1, IgG2, and IgM.

The samples were immediately placed on ice, followed by centrifugation (1,565 × *g*, 4°C, 20 min) and storage at -20°C until further analysis. The plasma metabolites were analyzed using an automatic spectrophotometer (ABX Pentra 400; Horiba ABX, Montpellier, France) and the respective kits: lactate (A11A01721), albumin (A11A01664) and triglycerides (A11A01640) from Horiba ABX (Montpellier, France); glucose (5530230), total protein (553–412), and cholesterol (553–127) from MTI-Diagnostics (Idstein, Germany; BHB; Randox Ranbut RB 1007); urea (LT UR0010) from Labor+Technik (E. Lehmann, Berlin, Germany); and NEFA (436 91995) from WAKO Chemicals, Neuss, Germany.

The plasma concentrations of IGF-I and insulin were determined using commercial immunoradiometric assays (IRMA IGF-I (A15729) and an insulin IRMA kit (IM3210; Immunotech s.r.o., Prague, Czech Republic) that were previously validated for bovine plasma [40,41]. The insulin-like growth factor-binding proteins in the plasma were analyzed using quantitative Western ligand blot analysis as described previously [42]. The IgG1 was measured using radial immunodiffusion with bovine reference serum (RS10-103; Bethyl Laboratories Inc., Montgomery, TX, USA) as a standard [43]; IgG2 and IgM using ELISA (no. E10-101 and no. E10-117, respectively; Bethyl Laboratories Inc.), fibrinogen using a rapid heat precipitation micromethod [43], and haptoglobin using the guaiacol method with human haptoglobin (Hp2-2; Sigma-Aldrich, Poznan, Poland) as a standard [44]. The detection limit of haptoglobin was 0.01 g/L.

### Liver Tissue Analyses

The liver tissue was crushed to a fine powder under liquid nitrogen using a mortar and pestle while snap frozen. The powdered tissue (50–100 mg) was homogenized using a FastPrep FP120A-230 (Thermo Electron Corporation, Milford, MA) cell disrupter system to extract total RNA using Trizol Reagent (Life Technologies, Darmstadt, Germany). The contamination of the extracted RNA with genomic DNA was removed using RNase-Free DNase (Qiagen GmbH, Hilden, Germany). The samples were purified using the RNeasy Mini Kit (Qiagen GmbH). The integrity and quality of total RNA were confirmed upon gel electrophoresis on denaturing agarose gels stained with ethidium bromide and after measuring the optical density at 260:280, which was 1.85 to 1.9, using a spectrophotometer (NanoPhotometer; Implen GmbH, Munich, Germany). For cDNA synthesis, 1 μg of RNA was reverse-transcribed using 200 U of the Reverse Transcriptase MMLV-RT RNase (H-) Point Mutant (Promega Corporation, Madison, WI) and 250 pmol random hexamer primers (Metabion International AG, Planegg-Steinkirchen, Germany). The obtained cDNA was diluted 1:4 with diethylpyrocarbonate (DEPC) water, and the aliquots were stored at -80°C. Specific primers were used to measure the mRNA expression of IGF 1 (*IGF1*), growth hormone receptor (*GHR*), IGF binding protein 1 (*IGFBP1*), *IGFBP-2*, *IGFBP-3*, *IGFBP-4*, insulin-like growth factor 1 receptor (*IGF1R*), insulin receptor (*INSR*), pyruvate carboxylase (*PC*), phosphoenolpyruvate carboxykinase, cytosolic isoform (*PCK1*) and mitochondrial isoform (*PCK2*), glucose-6-phosphatase (*G6PC*), propionyl-CoA carboxylase alpha chain, mitochondrial (*PCCA*), and solute carrier family 2, facilitated glucose transporter 2 (*SLC2A2*). The primers were designed using Primer-BLAST at NCBI (National Center for Biotechnology Information) or according to previous studies (Table 2), and real-time PCR was performed on a LightCycler 2.0 using FastStart DNA Master Plus SYBR Green I Master Mix (Roche Diagnostics GmbH, Mannheim, Germany) with 2 μL of cDNA. Each cDNA sample was analyzed in duplicate. To verify specific PCR products, a melting curve analysis was performed after the last amplification cycle. Furthermore, the product purity and size were confirmed using agarose gel electrophoresis, showing a single band at the expected size, followed by sequencing (ABI 3130 Genetic Analyzer (Life Technologies). The efficiency was calculated using LinRegPCR 2013 software [45]. Samples with an efficiency below 1.75 were discarded, and the preparation was repeated. The data calculation was performed using LightCycler analysis software 4.05 to evaluate the quantification cycles. The selection of appropriate reference genes and the quantification of the data were performed using qBase^+^ software (Biogazelle NV, Zwijnaarde, Belgium) [46]. Low-density lipoprotein receptor-related protein 10 (*LRP10*), emerin (*EMD*), and eukaryotic translation initiation factor 3 subunit K (*EIF3K*) served as reference genes.

**Table 2:** Characteristics of the Primers and PCR Conditions^1^.

Gene	Forward primer sequence 5’-3’	T_m_^2^ (°C)	Fragment length (bp)	T_a_^3^ (°C)	Source	GenBank accession no.
	Reverse primer sequence 5’-3’					
*LRP10*	CCAGAGGATGAGGACGATGT	60	139	60	[46]	BC149232.1
	ATAGGGTTGCTGTCCCTGTG	60				
*EMD*	GCCCTCAGCTTCACTCTCAGA	63	100	59	[47]	NM_203361
	GAGGCGTTCCCGATCCTT	58				
*EIF3K*	CCAGGCCCACCAAGAAGAA	59	125	58	[47]	NM_001034489
	TTATACCTTCCAGGAGGTCCATGT	64				
*IGF1*	TCGCATCTCTTCTATCTGGCCCTGT	65	240	62	[48]	NM_001077828.1
	GCAGTACATCTCCAGCCTCCTCAGA	66				
*GHR*	CCAGTTTCCATGGTTCTTAATTAT	61	138	56	[48]	NM_176608.1
	TTCCTTTAATCTTTGGAACTGG	61				
*IGFBP1*	TCAAGAAGTGGAAGGAGCCCT	61	127	57	[48]	NM_174554.3
	AATCCATTCTTGTTGCAGTTT	54				
*IGFBP-2*	CACCGGCAGATGGGCAA	57	142	57	[48]	NM_174555.1
	GAAGGCGCATGGTGGAGAT	59				
*IGFBP-3*	ACAGACACCCAGAACTTCTCCTC	65	193	58	[48]	NM_174556.1
	GCTTCCTGCCCTTGGA	63				
*IGFBP-4*	AAGATGAAGGTCATCGGGGC	60	150	60	this paper	NM_174557.4
	GCAGTTGGGGATGGGAATGA	60				
*IGF1R*	TTAAAATGGCCAGAACCTGAG	57	314	63	[48]	NM_001244612.1
	ATTATAACCAAGCCTCCCAC	56				
*INSR*	TCCTCAAGGAGCTGGAGGAGT	67	163	62	[49]	XM_590552
	GCTGCTGTCACATTCCCCA	68				
*PCK1*	Caaggatggggagccttgtg	60	122	60	[50]	NM_174737.2
	Cctccgaagatgatgccctc	63				
*PCK2*	Caactctcgcttttgtgccc	63	126	60	[50]	NM_001205594.1
	Gggggactcctttgggtcta	63				
*PC*	ACACCAACTACCCCGACAATG	66	353	60	[51]	AY185595
	CAGCGGGAGGTCAGGGAAG	69				
*G6PC*	ATGTTGTGGTTGGGATTCTGG	68	275	60	[51]	BC114011
	CACCTTCGCTTGGCTTTCTC	66				
*PCCA*	AACGTTTGGCAGCAGAAGAT	56	190	53	[52]	NM_001083509.1
	TGACAGGGTAGCCAATTTCC	58				
*SLC2A2*	ACAGAGGAATTGCCCACAAG	64	242	59	[51]	XM_614140
	TTCGAAAACCCCATCAAGAG	64				

*LRP10*, low-density lipoprotein receptor-related protein 10; *EMD*, emerin; *EIF3K*, eukaryotic translation initiation factor 3 subunit K; *IGF1*, insulin-like growth factor 1; *GHR*, growth hormone receptor; *IGFBP1*, insulin-like growth factor binding protein 1; *IGFBP-2*, insulin-like growth factor binding protein 2; *IGFBP-3*, insulin-like growth factor binding protein 3; *IGFBP-4*, insulin-like growth factor binding protein 4; *IGF1R*, insulin-like growth factor 1 receptor; *INSR*, insulin receptor; *PCK1*, phosphoenolpyruvate carboxykinase (cytosolic isoform); *PCK2*, phosphoenolpyruvate carboxykinase (mitochondrial isoform); *PC*, pyruvate carboxylase; *G6PC*, glucose-6-phosphatase; *PCCA*, propionyl-CoA carboxylase alpha chain, mitochondrial; *SLC2A2*, solute carrier family 2 facilitated glucose transporter member 2.

^1^Conditions for each cycle: Denaturation (95°C, 15 s), annealing (distinct T_a_ as listed above, 10 s), extension (72°C, 30 s)

^2^T_m_, melting temperature of the primer

^3^T_a_, annealing temperature

The glycogen and glucose concentrations in the liver were determined using an enzyme-based starch-kit (no. 10207748035; Roche Diagnostics GmbH, Mannheim, Germany) with wet tissue (25 mg) frozen under liquid nitrogen and then subjected to a mortar and pestle while snap frozen.

### Statistical Analyses

All results are presented as the least squares means with the standard error. The data analysis was generated using SAS/STAT software, Version 9.3 of the SAS System for Windows (SAS Institute Inc., Cary, NC, USA). The data of total dry mater intake, feed efficiency, from liver tissue, days of scours, and carcasses were evaluated with the General Linear Model (GLM) procedure with group (feeding intensity), breed, and gender as the main effect. The data on feed intake, growth performance, and measurements in the blood plasma were evaluated using the MIXED procedure of SAS with group (MR feeding intensity), time (day/week of life), group × time interaction, breed, and gender as fixed effects. Repeated measures on the same calf were considered using the REPEATED statement of the MIXED procedure, an unstructured type for the block diagonal residual covariance matrix selected for the plasma data and an autoregressive (1) type for the performance data. Individual differences in both models were examined using the Tukey-Kramer method, and the data were considered significant with *P* < 0.05 and as a trend with *P* < 0.1. The effects of breed and gender were included in the final model only when significant (*P* < 0.05). The F-values and the degree of freedom were in addition reported. In the Mixed Model, the interactions were evaluated based on the slice statement for the partitioned analysis of the least squares means.

## Results

### Feeding, Growth Performance, and Health Status

**Fig 1:**
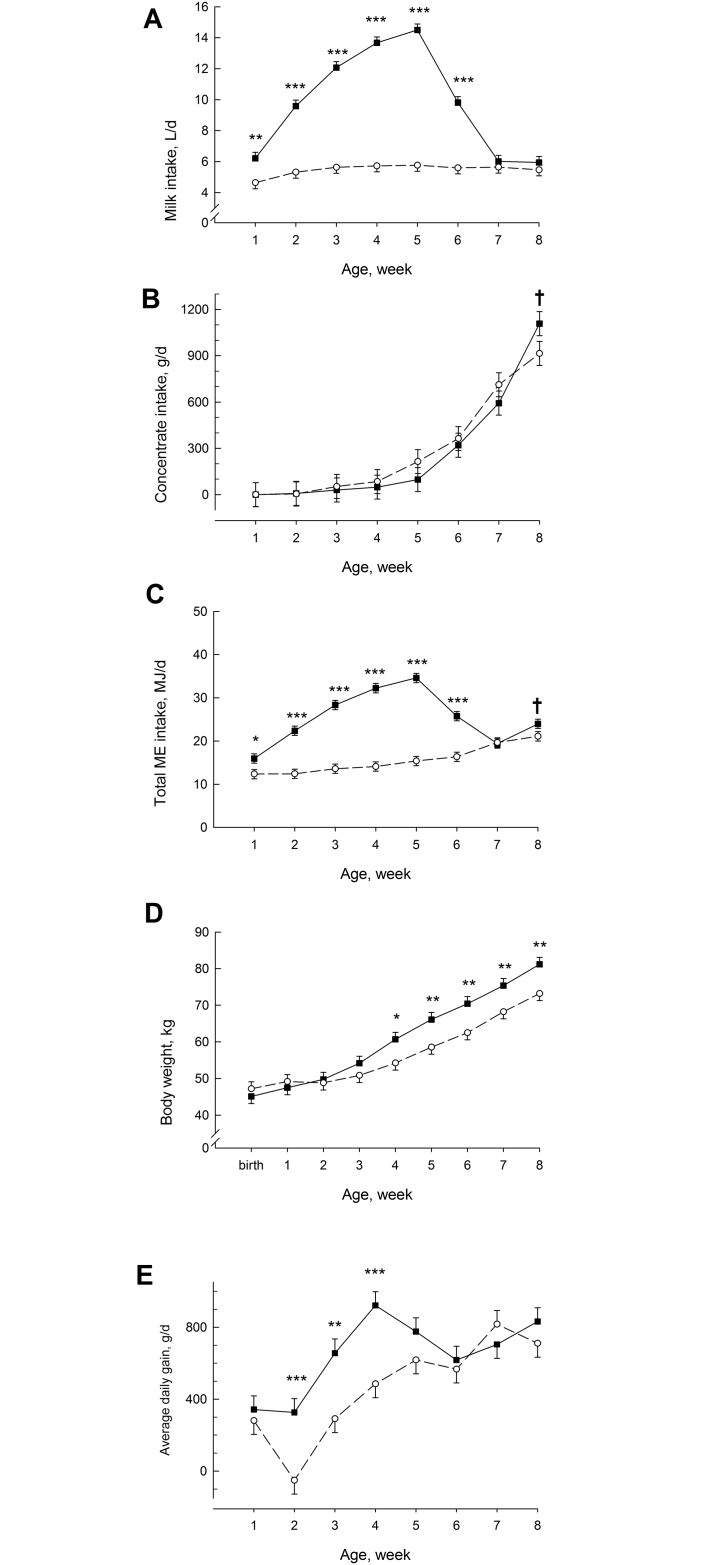
Feed Intake and Growth Performance. Colostrum and milk replacer (MR) (**A**) and concentrate intake (**B**), total metabolizable energy (ME) intake, body weight (**D**), and average daily gain (**E**) in calves fed MR either *ad libitum* (ADLIB, black square, straight line) or restrictively (RES, white circle, dashed line) for the first five weeks of life. MR intake in ADLIB was gradually reduced during weeks 6 and 7 to amounts fed in RES. The data are presented as the least squares means ± standard error. ^†^Trend between groups *P* < 0.1; *different between groups *P* < 0.05; **different between groups *P* < 0.01; and ***different between groups *P* < 0.001.

MR intake increased (F_7,178_ = 33.4; *P* < 0.001; Fig 1A) in ADLIB from week 1 to week 5 and decreased thereafter, reaching the MR intake level of RES. The MR intake was greater (F_1,56_ = 243; *P* < 0.001) in ADLIB than in RES from week 1 to week 6. Concentrate intake increased (F_7,164_ = 39.6; *P* < 0.001) with time in both groups and tended to be greater at the end of the study in ADLIB than in RES (F_1,53_ = 3.09; *P* = 0.08; Fig 1B). Mean total dry matter intake increased in ADLIB and RES from 0.85 ± 0.03 kg/d and 0.66 ± 0.03 kg/d in the first week to 1.72 ± 0.13 kg/d (F_7,20_ = 49.4; P < 0.001) and 1.49 ± 0.13 kg/d (F_7,20_ = 8.24; P < 0.001) in the last week of the study. Highest dry matter intake in ADLIB was determined in week 5 (1.90 ± 0.06 kg/d) and in RES at the end of the study. Dry matter intake was greater (F_1,26_ = 64.7; P < 0.001) in ADLIB than in RES with respect to the total experimental period. ME intake increased (F_7,175_ = 32.0; P < 0.001; Fig 1C) in ADLIB from week 1 to week 5 and decreased thereafter, reaching the ME intake level of RES at week 7. In RES, ME intake increased (F_7,175_ = 6.07; P < 0.001) continuously from week 1 to week 8. The ME intake was greater (F_1,146_ = 243; *P* < 0.001) from week 1 to week 6 and tended to be greater (F_1,146_ = 3.5; *P* < 0.1) at week 8 in ADLIB than in RES. The sum of total dry matter and MR intake (dry matter) as well as ME intake were greater (F_1,27_ = 64.9; P < 0.001, F_1,27_ = 203; P < 0.001, F_1,27_ = 109; P < 0.001) in ADLIB than in RES, but sum of concentrate intake (dry matter) did not differ between groups (Sum of total dry matter, milk dry matter, ME, and concentrate dry matter intake for 8 weeks were 82.3 ± 2.5 kg, 66.7 ± 1.5 kg, 1418 ± 36 MJ, and 13.6 ± 1.9 kg for ADLIB and 53.3 ± 2.5 kg, 38.9 ± 1.5 kg, 874 ± 36 MJ, and 14.5 ± 1.9 kg for RES). Gender and breed did not affect feed intake at all.

The body weight at birth was similar between the groups, increasing in both groups (F_8,206_ = 128; *P* < 0.001), and was greater (F_1,34_ = 5.62; *P* < 0.001; Fig 1D) in ADLIB than in RES from week 4 on. Average daily weight gain indicated time changes (F_7,166_ = 18.3; *P* < 0.001; Fig 1E), decreased from the 1st to 2nd week in RES calves, and increased thereafter in both groups during the 1st month of life. The increase from week 2 to week 4 was greater (F_1,207_ = 12.2; *P* < 0.001) in ADLIB than in RES. During the entire experimental period feed efficiency, namely body weight gain/dry matter intake, body weight gain/ME intake, and body weight gain/protein intake were greater (F2,27 = 5.7; P < 0.05, F2,27 = 12; P < 0.01, F2,27 = 7.9; P < 0.01) in RES than in ADLIB (body weight gain/dry matter intake, body weight gain/ME intake, and body weight gain/protein intake for 8 weeks were 0.47 ± 0.02 kg/kg, 27.2 ± 1.1 g/MJ, and 43.6 ± 1.7 g/kg for ADLIB and 0.53 ± 0.02, 31.8 ± 1.1 g/MJ, and 49.8 ± 1.8 g/kg for RES). Feed efficiency was greater (F2,27 = 13.4; P < 0.01, F2,27 = 10.9; P < 0.01, F2,27 = 12.6; P < 0.01 for body weight gain/dry matter intake, body weight gain/ME intake, and body weight gain/protein intake) in Holstein than in Holstein x Charolais calves.

Number of days treated for diarrhea was similar in both groups (ADLIB 3.71 ± 0.94 d, RES 1.86 ± 0.94 d). At harvest, the carcass weight and perirenal fat mass were greater (F_1,27_ = 6.5; *P* < 0.05 for carcass; (F_1_ = 10.3; *P* < 0.001 for perirenal fat; Table 3) and liver weight tended to be greater (F_1,27_ = 2.94; *P* < 0.1) in ADLIB than in RES. The muscle mass in most cuts and the size of the musculus longissimus dorsi were greater (F_1,27_ = 4.65; *P* < 0.05; Table 3) in ADLIB than in RES. The breed and gender barely affected the carcass composition. The mass of the hind shank and brisket was greater (F_1_ = 5.15; *P* < 0.001 for hind shank; F_1_ = 9.86; *P* < 0.001 for brisket) in Holstein x Charolais than in Holstein calves, while the mass of the perirenal fat was greater (F_1_ = 4.88; *P* < 0.05) in female than in male calves.

**Table 3:** Carcass Composition. The weight of the hot carcass, liver, pancreas, fat depots, and distinct muscle cuts^1^ and the size of the musculus longissimus dorsi (MLD) in calves fed milk replacer (MR) either *ad libitum* (ADLIB) or restrictively (RES) for the first five weeks of life.

Item	Feeding intensity^2^		SE	*P*-value
	ADLIB	RES		
Hot carcass, left side (kg)	22.42	20.24	0.60	0.02
Liver (kg)	1.50	1.38	0.05	0.10
Pancreas (g)	83.57	79.29	4.98	0.55
Perirenal fat (kg)	0.45	0.27	0.04	< 0.01
Hind shank (kg)^3^	0.76	0.66	0.02	< 0.01
Round (kg)^3^	5.43	4.74	0.16	< 0.01
Sirloin (kg)^3^	1.04	0.89	0.04	< 0.01
Tenderloin (kg)^3^	0.47	0.40	0.02	< 0.01
Flank (kg)^3^	0.66	0.58	0.02	< 0.01
Skirt (kg)^3^	0.43	0.35	0.02	< 0.01
Chuck back rib (kg)^3^	1.21	1.08	0.05	0.05
Neck (kg)^3^	1.43	1.24	0.06	0.04
Short plate (kg)^3^	0.63	0.58	0.02	0.11
Brisket (kg)^3^	0.78	0.58	0.04	< 0.01
Boned shoulder (kg)^3^	2.06	1.85	0.07	0.03
Fore shank (kg)^3^	0.48	0.41	0.02	< 0.01
Sum of muscle (kg)	12.23	10.68	0.37	< 0.01
MLD length (cm)	50.00	47.86	0.70	0.04
MLD circumference (cm)	19.71	18.57	0.38	0.04

Data presented as least squares means ± standard error.

^1^Muscle cuts are described in [40].

^2^Calves fed MR either *ad libitum* (ADLIB) or restrictively (RES) for the first five weeks of life. MR intake in ADLIB was gradually reduced during weeks 6 and 7 to amounts fed in RES.

^3^ Cuts are illustrated in S1 Fig.

### Metabolic and Endocrine Changes in the Blood Plasma

**Fig 2:**
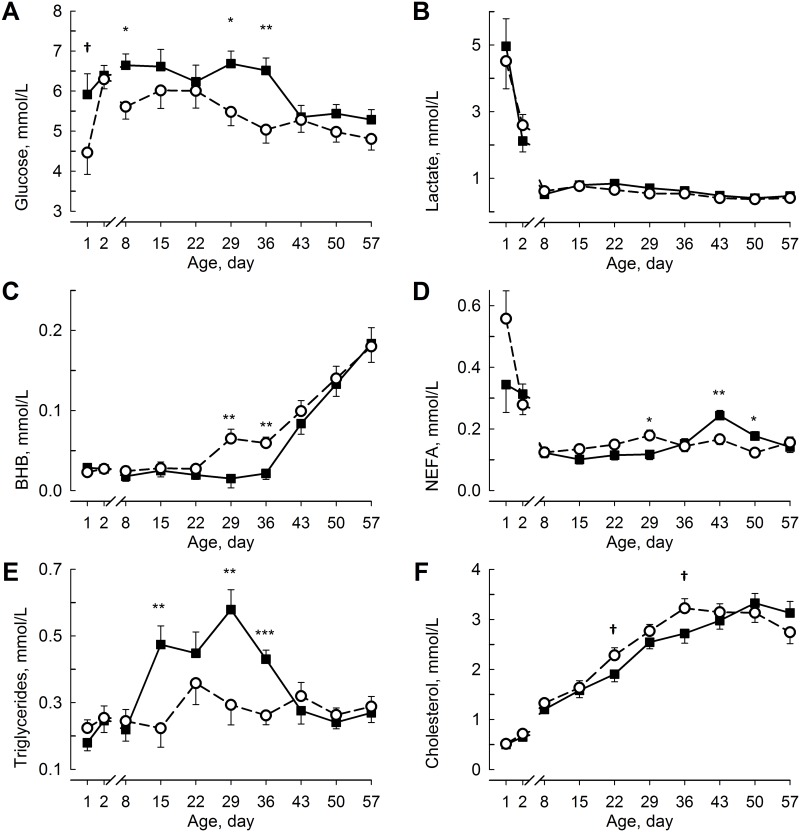
Metabolic Status in Blood Plasma. The plasma concentrations of glucose (**A**), lactate (**B**), beta-hydroxybutyrate (BHB; **C**), non-esterified fatty acids (NEFA; **D**), triglycerides (**E**), and cholesterol (**F**) in calves fed milk replacer (MR) either *ad libitum* (ADLIB, black square, straight line) or restrictively (RES, white circle, dashed line) for the first five weeks of life. MR intake in ADLIB was gradually reduced during weeks 6 and 7 to amounts fed in RES. The data are presented as the least squares means ± standard error. ^†^Trend between groups *P* < 0.1, *different between groups *P* < 0.05, **different between groups *P* < 0.01, and ***different between groups *P* < 0.001.

At birth, the plasma glucose concentration was lower (F_1,24.7_ = 3.99; *P* < 0.1; Fig 2A) in RES than in ADLIB, but the glucose concentration increased (F_9,17.8_ = 6.25; *P* < 0.001) at 24 h after birth to the same concentration in both groups. The plasma glucose concentration decreased from day 22 on in RES and from day 36 on in ADLIB, respectively. Plasma glucose was greater (F_1,24.6_ = 7.33; *P* < 0.05 for day 8) in ADLIB than in RES on days 8, 29, and 36. The plasma glucose concentration was greater (F_1,21.2_ = 6.22; *P* < 0.05) in female than in male calves and was greater (F_1,20.6_ = 11.75; *P* < 0.05) in Holstein than in Holstein x Charolais calves. The plasma lactate concentration decreased (F_9,18_ = 10.1; *P* < 0.001; Fig 2B) in both groups during the first week of life but remained stable thereafter and did not show group differences. The plasma BHB concentration increased (F_9,18_ = 19.9; *P* < 0.001; Fig 2C) in RES after 22 days and in ADLIB after 36 days of life. The plasma BHB concentration was greater (F_1,25.3_ = 8,39; *P* < 0.01 for day 29) in RES than in ADLIB at 29 and 36 days, respectively. The plasma NEFA concentration decreased (F_9,18_ = 14.8; *P* < 0.05; Fig 2D) during the 1st week of life in both groups and was lower (F_1,25.9_ = 6.24; *P* < 0.05) on day 28, but was greater (F_1,25.3_ = 10.6 for day 43; *P* < 0.05) on days 43 and 50 in ADLIB than in RES. The plasma NEFA concentration tended to be greater (F_1,24_ = 3.82; *P* < 0.1) in male than in female calves and was greater (F_1,24_ = 18.9; *P* < 0.05) in Holstein than in Holstein x Charolais calves. The plasma triglyceride concentration increased (F_9,18_ = 10.9; *P* < 0.001; Fig 2E) in ADLIB after the first week of life and was greater (F_1,26.1_ = 10.1 for day 15; *P* < 0.01) from 15 to 36 days in ADLIB than in RES. In both groups, from day 43 on, the triglyceride concentration decreased to the concentration observed in the 1st week of life. The plasma triglyceride levels were greater (F_1,24_ = 6.18; *P* < 0.05) in Holstein than in Holstein x Charolais calves. The plasma cholesterol concentration increased (F_9,17.3_ = 84.8; *P* < 0.001; Fig 2F) in both groups, reaching the highest concentrations in RES on day 36 and in ADLIB on day 50. The cholesterol concentration tended to be lower (F_1,25.5_ = 3.34; *P* < 0.1 for day 22) in ADLIB than in RES on days 22 and 36.

**Fig 3:**
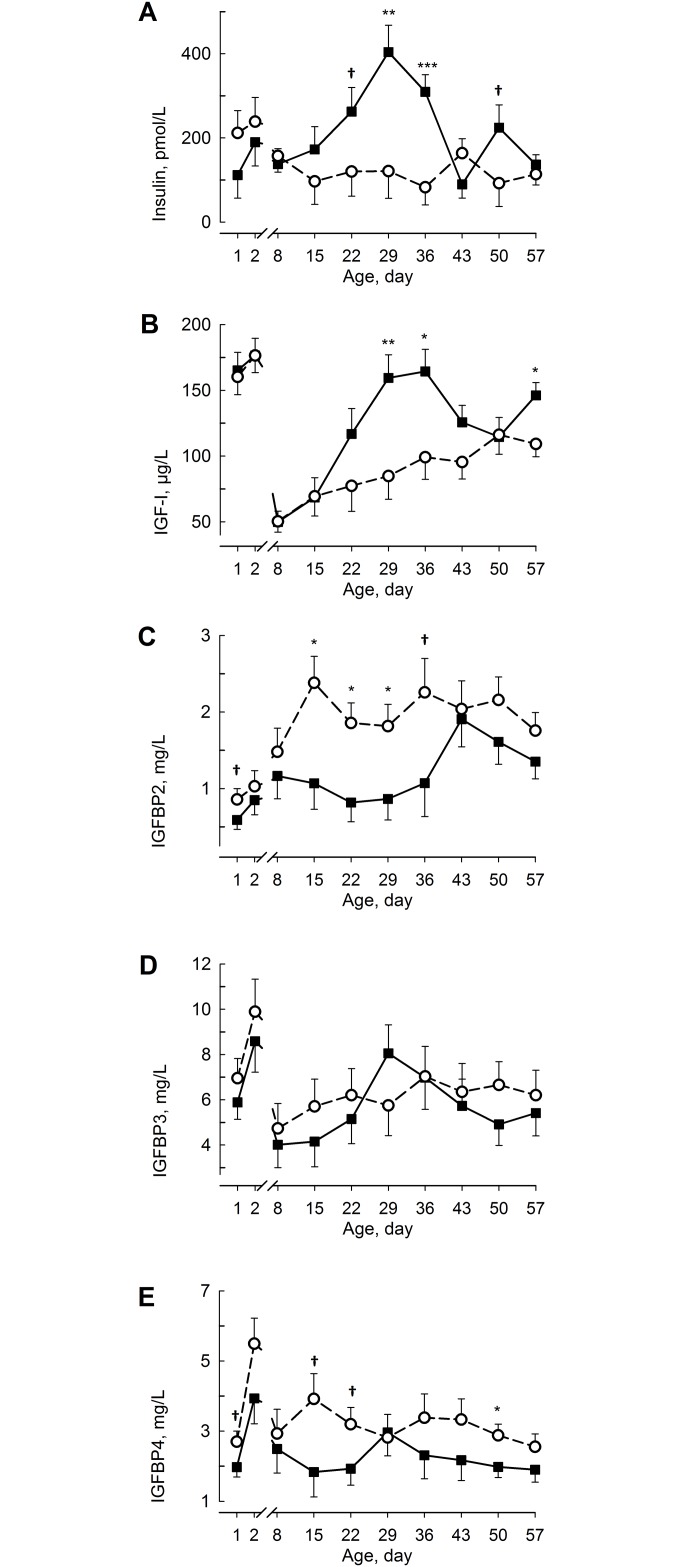
Endocrine Status in Blood Plasma. The plasma concentrations of insulin (**A**), insulin-like growth factor 1 (IGF-I; **B**), and IGF-binding proteins (IGFBP)-2 (**C**), -3 (**D**), and -4 (**E**) in calves fed milk replacer (MR) either *ad libitum* (ADLIB, black square, straight line) or restrictively (RES, white circle, dashed line) for the first five weeks of life. MR intake in ADLIB was gradually reduced during weeks 6 and 7 to amounts fed in RES. The data are presented as the least squares means ± standard error. ^†^Trend between between groups *P* < 0.1, *different between groups *P* < 0.05, **different between groups *P* < 0.01, and ***different between groups *P* < 0.001.

The plasma insulin concentration increased (F_9,17.2_ = 3.19; *P* < 0.05 for the group x time interaction; Fig 3A) after birth in ADLIB and showed the highest concentration after 29 days of life but did not change with time in RES calves. The insulin concentration was greater on days 29 (F_1,25.7_ = 9.95; *P* < 0.001) and 36 (F_1,26_ = 16.2; *P* < 0.001) and tended to be greater on days 22 (F_1,24.5_ = 3.14; *P* < 0.1) and 50 (F_1,25.4_ = 3.00; *P* < 0.1) in ADLIB than in RES. The plasma insulin concentration was greater (F_1,23.3_ = 6.78; *P* < 0.05) in female than in male calves. The plasma IGF-I concentration decreased (F_9,17.7_ = 34.4; *P* < 0.001; Fig 3B) during the 1st week of life but increased thereafter in both groups. The plasma IGF-I concentration was greater (F_1,23.8_ = 9.03; *P* < 0.05 for day 29) on days 29, 36, and 57 in ADLIB than in RES. The plasma IGFBP-2 concentration increased (F_9,18_ = 4.19; *P* < 0.001; Fig 3C) in both groups; showing highest concentration in RES on day 15 and in ADLIB on day 43. The IGFBP-2 concentration was greater (F_1,25.8_ = 7.68; *P* < 0.05 for day 15) from day 15 until day 29 and tended to be greater (F_1,26_ = 3.73; *P* < 0.1 for day 36) on days 1 and 36 in RES than in ADLIB. The plasma IGFBP-3 concentration increased (F_9,18_ = 4.88; *P* < 0.001; Fig 3D) at 24 h after birth but decreased to day 8 in both groups. The plasma IGFBP-4 concentration increased (F_9,18_ = 6.08; *P* < 0.01; Fig 3E) during the first 24 h but subsequently decreased until day 8 in both groups. In RES, the IGFBP-4 concentration increased, whereas in ADLIB IGFBP-4 decreased on day 15. The IGFBP-4 concentration tended to be lower (F_1,26_ = 3.51; *P* < 0.1 for day 15) on days 1, 15, and 22 and was lower (F_1,26.1_ = 4.86; *P* < 0.05) on day 50 in ADLIB compared with RES. For all 3 IGFBPs, the plasma concentrations were greater (F_1,24_ = 5.44, 9.61, and 10.4 for IGFBP-2, -3, and -4; *P* < 0.05, respectively) in female than in male calves.

**Fig 4:**
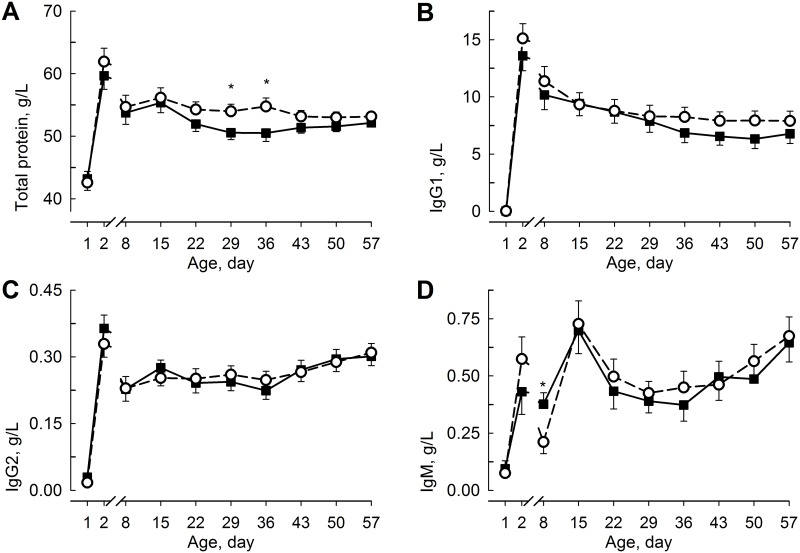
Total Protein and Immune Status in Blood Plasma. Plasma concentrations of total protein (**A**) and immunoglobulins (Ig) G1 (**B**), IgG2 (**C**), and IgM (**D**) in calves fed milk replacer (MR) either *ad libitum* (ADLIB, black square, straight line) or restrictively (RES, white circle, dashed line) for the first five weeks of life. MR intake in ADLIB was gradually reduced during weeks 6 and 7 to amounts fed in RES. The data are presented as the least squares means ± standard error. *Different between groups *P* < 0.05.

The plasma concentrations of total protein, IgG1, IgG2, and IgM increased (F_9,17.8_ = 29.1; F_9,232_ = 85.6; F_9,16.6_ = 35.5; F_9,17.5_ = 7.28 for total protein, IgG1, IgG2, and IgM; *P* < 0.001; Fig 4A–4D) after the 1st colostrum intake and decreased to day 8. Thereafter, the total protein and IgG2 concentrations remained constant, while the IgG1 concentration slowly decreased, and the IgM concentration was highest on days 15 and 57 in both groups. The plasma concentration of total protein was greater (F_1,25.9_ = 5.07; *P* < 0.05 for day 29) on days 29 and 36 in RES than in ADLIB and was greater (F_1,23.1_ = 18.0; *P* < 0.05) in female than in male calves. The plasma concentrations of IgG1 and IgG2 did not differ between groups, whereas the IgM concentration on day 8 was greater in ADLIB than in RES (F_1,21.9_ = 4.48; *P* < 0.05).

**Fig 5:**
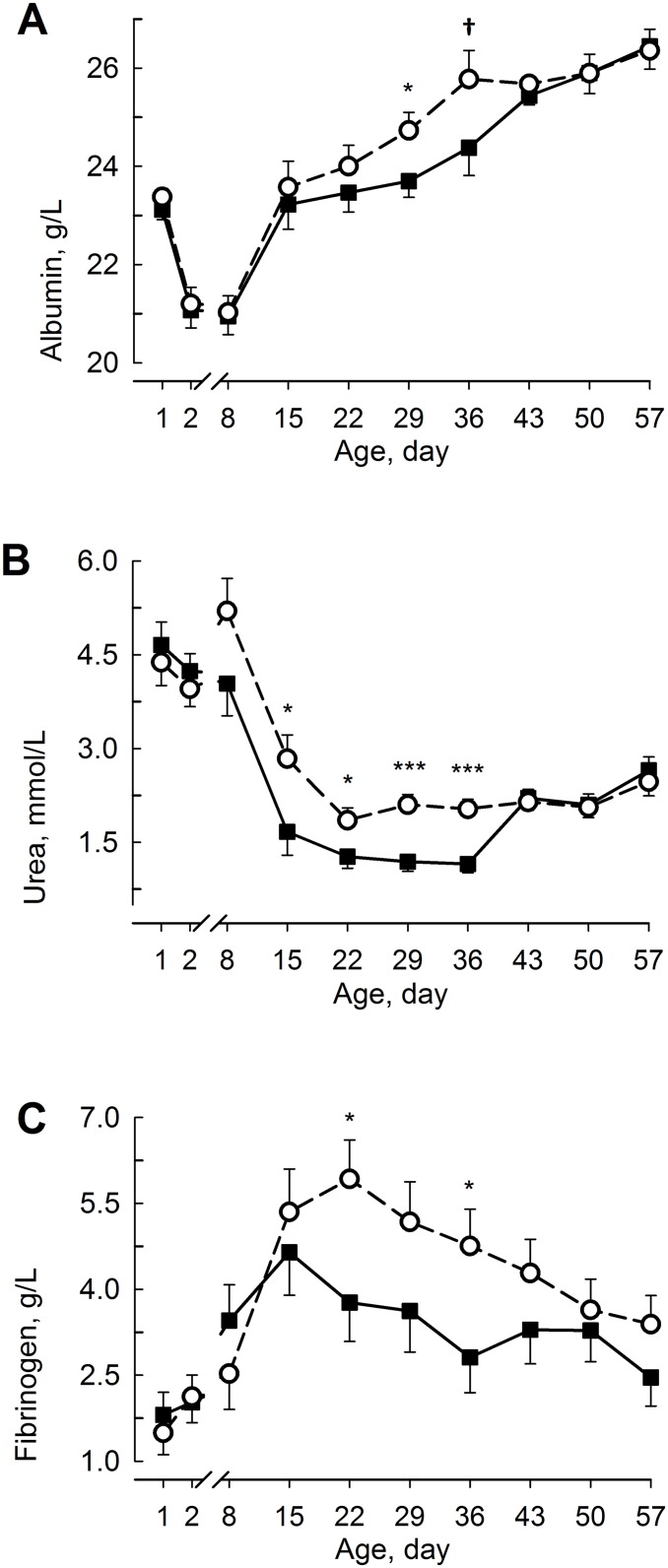
Inflammatory Status and Urea in Blood Plasma. The plasma concentrations of albumin (**A**), urea (**B**), and fibrinogen (**C**) in calves fed milk replacer (MR) either *ad libitum* (ADLIB, black square, straight line) or restrictively (RES, white circle, dashed line) for the first five weeks of life. MR intake in ADLIB was gradually reduced during weeks 6 and 7 to amounts fed in RES. The data are presented as the least squares means ± standard error. ^†^Trend between between groups *P* < 0.1, *different between groups *P* < 0.05, and ***different between groups *P* < 0.001.

The plasma albumin concentration decreased (F_9,18.1_ = 60.9; *P* < 0.001; Fig 5A) during the 1st week of life and slowly increased thereafter until day 57. The plasma concentration was greater (F_1,23.6_ = 5.52; *P* < 0.05) on day 29 and tended to be greater (F_1,26.3_ = 3.23; *P* < 0.1) on day 36 in RES than in ADLIB. Moreover, the plasma concentration was greater (F_1,24.3_ = 9.96; *P* < 0.05) in female than in male calves. The plasma urea concentration decreased in ADLIB until day 15, whereas in RES, the urea concentration increased on day 8 and decreased thereafter until day 22 (F_9,18_ = 20.1; *P* < 0.001; Fig 5B). The urea concentration was lower (F_1,25.8_ = 5.02; *P* < 0.05 for day 15) in ADLIB than in RES from day 15 until day 36 and was lower (F_1,24_ = 5.81; *P* < 0.05) in Holstein than in Holstein x Charolais calves. The plasma fibrinogen concentration increased (F_9,15.3_ = 3.84; *P* < 0.001; Fig 5C) in both groups after birth and was highest in ADLIB on day 15 and in RES on day 22. Furthermore, the fibrinogen concentration was greater (F_1,24.8_ = 5.15; *P* < 0.05 for day 22) in RES than in ADLIB on days 22 and 36. Haptoglobin was detected only in 6 out of the 252 plasma samples examined. Therefore, no statistics were performed. In one ADLIB calf, the haptoglobin concentrations increased on day 15 to 0.66 g/L.

### Hepatic Gene Expression and Glycogen Concentration

Expression of hepatic mRNA was comparable between ADLIB and RES, except for *PCK1*, which was greater (F_1,27_ = 4.66; *P* < 0.05) in ADLIB than in RES (1.25 versus 0.92 ± 0.11 relative to reference genes). The liver glycogen (3.05 versus 3.58% of wet weight ± 0.41, *P* = 0.37) and liver glucose (1.48 versus 1.35% of wet weight ± 0.12, *P* = 0.43) concentrations were comparable between ADLIB and RES. In addition, breed and gender had no effect on the parameters measured in the liver.

## Discussion

Feeding unlimited amounts of MR to calves during the first five weeks of life resulted in more than twice as much MR intake in ADLIB than in RES receiving only 6 liters of MR per day, an elevated average daily gain during the intensive MR feeding period, increased body weight until the end of the study period, and increased skeletal muscle and perirenal fat growth. Our data on milk intake in ADLIB calves were in the range with data from other studies with *ad libitium* milk feeding using an automate or by sucking [10,13,53], but not when feeding milk *ad libitum* by bucket [11]. In addition, MR intake was much greater in ADLIB calves than in studies with intensive milk feeding programs when there was a time limit for milk access [54], or restricted amounts of MR were fed with elevated nutrient content [5,7,8]. In contrast to Maccari et al. [13] calves in the present study received milk *ad libitum* for 5 weeks, resulting in a greater dry matter and ME intake herein during the first 5 weeks of life. Average daily gain reflects milk feeding intensity in pre-weaning calves, as seen in this study, but decreases when intensive milk feeding stops [13]. This finding demonstrates that solid feed intake cannot immediately compensate for the reduction in milk intake, probably because there is a delay in the increase of solid feed intake when the intensive milk feeding program stops [9,13]. An intensive MR-feeding program enhances the protein content in the carcass, although the protein percentage in the carcass decreases and the relative fat mass increases with elevated MR feeding [5,7]. However, intensive MR-fed calves catabolize less amino acids, as indicated by the reduced plasma urea concentration in intensive milk-fed calves [5], a finding consistent with the present study. In contrast to previous studies with intensive milk feeding programs in pre-weaning calves [5,12,16], the overall feeding efficiency in the present study was slightly lower in ADLIB than in RES calves. The reason for this divergent finding is presently not known, but probably the *ad libitum* feeding schedule in the present study caused the impaired feeding efficiency, because milk intake was not *ad libitum* in the previous studies [5,12,16].

Calves fed *ad libitum* amounts of MR consumed and apparently digested high amounts of nutrients without detrimental effects on gastrointestinal function, such as indigestion or diarrhea [11–13]. Recent studies in milk-fed calves have indicated that the abomasum of 3-week old calves increases in size during milk feeding and can manage a milk portion greater than 2–3 liters [55]. In addition, automated milk feeding facilitates the division of the daily milk ration into several portions, and calves provided milk *ad libitum* drink their daily ration in approximately 6 portions [10]. On the other hand, concentrate or solid feed intake in calves is often reduced during the intensive milk feeding program [9,11,19,21,56], but this observation has not been reported in all calf studies with an intensive milk feeding program [12,13]. The concentrate intake in the present study with respect to the whole experimental period was not affected by *ad libitum* milk feeding and was in the range observed in a previous study [12,13]. More frequent milk feeding with smaller milk portions during the day might facilitate concentrate intake in pre-weaned calves.

The plasma concentrations of total protein and immunoglobulins indicated a sufficient supply of immunoglobulins, as the plasma levels were beyond the critical level for the failure of passive immunoglobulin transfer [43,57]. Interestingly, the plasma IgM concentration showed a second peak in plasma after the first weeks of age and this peak may reflect antigenic stimulation by environmental antigens. Recent findings in colostrum-deprived calves showed that calves exhibit indigenous IgM production during the first week of life [58]. Although statistically not significant, the lower plasma IgG1 concentration in ADLIB between days 36 and 57 indicates that antigenic stimulation in this group was on a lower level and the risk for infection or antigenic stimulation was obviously reduced in this group after first month of life.

The elevated plasma concentration of total protein at the end of the first month in RES likely results from greater plasma concentrations of albumin and fibrinogen. Fibrinogen and haptoglobin are acute phase proteins and reliable markers for local and systemic inflammation. The plasma concentrations of these proteins increase during inflammation [59,60]. Greater plasma fibrinogen concentrations in RES might suggest a slightly increased incidence of inflammatory events in these calves. This finding is consistent with the study of Obeidat et al. [61], who showed greater neutrophil responses in calves with a low plane of nutrition prior to weaning, and supports the conclusions of Khan et al. [9], who recommended increased milk intake to improve the health status of pre-weaned calves. However, number of calves in this study was too low to evaluate the health status and and plasma haptoglobin was barely detectable. Plasma haptoglobin concentrations below 0.1 g/L are usually observed in clinically healthy calves [62]. In the present study one ADLIB calf with elevated plasma haptoglobin had loose feces during second week of life and possibly suffered from a bacterial infection [62]. An increased haptoglobin concentration was recently measured only in colostrum-deprived calves during first week of life [58].

Furthermore, the plasma albumin concentration typically decreases during the acute phase response in calves, as albumin is a major negative acute phase protein [60]. The lower plasma albumin concentration in ADLIB calves was likely not an inflammatory response but may indicate a greater dilution effect on plasma albumin during the enhanced MR feeding program and increased liquid intake. We have previously shown a decreased postprandial albumin concentration after colostrum feeding in neonatal calves when compared to formla-fed calves [63,64]. The greater plasma concentrations of total protein and fibrinogen may partly also result from a hemo-diluting effect in ADLIB due to a greater MR intake [62].

The plasma metabolite concentrations clearly indicated an improved nutritional status in ADLIB during the intensive MR feeding period. In addition, both the plasma glucose and plasma triglyceride concentrations were greater during intensive MR feeding in ADLIB, consistent with previous findings [10,12,13]. In general, the plasma glucose decreases with age and increasing ruminant function [32,65]. Khan et al. [12] reported an elevated plasma glucose concentration after weaning in intensive milk-fed calves during the first four weeks of life, likely reflecting elevated propionate production in the rumen and hepatic gluconeogenesis [66]. The calves in the present study were not weaned. Although we did not observe increased rumen activity in ADLIB calves after the step-down period, indicated by a similar BHB concentration in the plasma from days 43 to 57 in ADLIB and RES, the increased hepatic mRNA expression of *PCK1*, encoding a key enzyme of hepatic gluconeogenesis, might suggest higher hepatic gluconeogenic activity at the end of the study period in ADLIB. In calves, hepatic *PCK1* gene expression increases with age [67], and the hepatic enzyme activity of phosphoenolpyruvate carboxylase is positively associated with gluconeogenesis [68]. However, the plasma glucose levels were not significantly different at the end of the study period.

The plasma NEFA concentrations were lower during the intensive MR feeding period, indicating less body fat mobilization in ADLIB. However, the plasma NEFA was slightly increased during the step-down period in ADLIB, assuming that some body fat mobilization occurred. The increased plasma BHB is an indicator for the onset of rumen function in milk-fed calves [65,66,69]. Although the concentrate intake was not different, the digestion and absorption of nutrients in the rumen were slightly lower in ADLIB than RES calves at the end of *ad libitum* MR feeding, as plasma BHB was lower in ADLIB than in RES calves at that time. The plasma cholesterol concentration increased, consistent with previous studies [10,13], was greater in RES than in ADLIB. This result suggested an elevated synthesis or reduced metabolism of cholesterol in RES calves, as the cholesterol intake was lower in RES in response to lower MR intake.

Changes in the plasma concentrations of insulin, IGF-I, and IGF binding proteins 2 and 4 during the intensive MR-feeding period correlated with the enhanced protein and energy intake in ADLIB calves and supported previous findings on the plasma insulin and IGF-I concentrations in calves [7,13,31–33,70]. The maturation of the postnatal somatotropic axis depends on the nutritional supply, initiated during the postnatal period, and reflects the glucose status and insulin action [28,35,71,72]. Although we have not determined the GH status in the present study, the enhanced plasma concentrations of glucose and insulin have most likely caused the accelerated maturation of the somatotropic axis in ADLIB, consistent with the increased IGF-I plasma concentration during the intensive milk-fed period [26,71,72]. Interestingly, the plasma IGF-I decreased during the step-down of MR feeding in ADLIB but again was greater in ADLIB than in RES at the end of the study period. At that time, the MR intake was the same, whereas the concentrate intake was greater in ADLIB calves. Because nutrient digestion in the rumen was significant at the end of the study period, evident from the increasing BHB concentration in the plasma of both groups, the increased concentrate intake might provide enough protein and energy to stimulate rumen development and plasma IGF-I, as previously described [7]. In goats, the energy intake affects rumen development and local and systemic IGF-I expression [73], while in steers, plasma IGF-I is regulated through protein and energy intake [74]. The elevated muscle growth observed in the carcass at the end of the study period was associated with the enhanced IGF-I and insulin status in ADLIB, as both IGF-I and insulin favor muscle growth [29,75], and the effects of the somatotropic axis on muscle growth depend on the feeding level [76].

IGF binding proteins are important regulators of IGF-I action, and the inhibitory and stimulating effects of IGFBPs have been reported [28,38]. The increase in plasma concentrations of IGFBP-3 and -4 on day 2 of life probably does not reflect the ingestion of colostral IGFBPs, because in previous studies, no absorption of colostral IGFBP or IGF-I was observed during the first day of life [24–26,35,77]. The plasma IGFBP-2 concentration shows an inverse relationship with respect to the insulin and IGF-I status in calves and dairy cows [24,32,78] and is elevated during the catabolic state [28,37]. Therefore, increasing plasma IGFBP-2 may reflect the inadequate nutrient supply in RES and the impaired nutrient status in ADLIB during the step-down period. Time changes in the plasma IGFBP-3 concentration were small, and surprisingly, no feeding effect was observed during the intensive MR-feeding period. Previous studies have shown an increase in plasma IGFBP-3 in calves with age [79,80] and MR-feeding intensity [32], and plasma IGFBP-3 normally follows plasma IGF-I, because IGFBP-3, together with an acid-labile subunit, binds most of the IGF-I in circulation and therefore correlates well with IGF-I [28,37,38]. In addition, the plasma IGFBP-3 inversely correlates with IGFBP-2 during growth [29,37], which was also not observed in the present study. The reasons for this finding are presently unknown, but studies in bulls suggested that IGFBP-3 is likely not a good indicator of postnatal growth [81,82]. However, similar to IGFBP-2, plasma IGFBP-4 was lower in ADLIB than in RES, even after the cessation of free MR intake in ADLIB. Both IGFBP-2 and -4 exhibit IGF-I inhibitory effects [28,38]. Thus, lower plasma IGFBP-2 or -4 may indicate reduced growth stimulation in RES. However, in a previous study, intensive MR-feeding had no effect on plasma IGFBP-4 [32].

Contrary to the systemic diet effects on the IGF system, the hepatic gene expression of the somatotropic axis was not affected at the end of the study period in response to intensive MR feeding during the first five weeks of life. There is a good correlation between the gene expression of the hepatic somatotropic axis and circulating IGF-I, because the liver produces much of the systemic IGF-I and IGFBPs [38,70]. However, elevated plasma IGF-I was obviously not an effect of enhanced hepatic IGF-I gene expression, but other tissues, such as muscle, might contribute to the elevated plasma IGF-I observed in ADLIB [83]. The lack of differences in the hepatic somatotropic axis reflects the comparable protein and energy intake in both groups at the end of the study. Because the IGF-I gene expression is regulated by the protein and energy intake no differences should be expected [84]. On the contrary, recent findings in calves indicate increased IGF-I mRNA abundance in the liver during intensive MR feeding and greater protein and energy intake (D. Frieten, C. Koch, H.M. Hammon, unpublished observation).

Breed and gender effects are not the main topic of the present study, as the distribution occurred randomly and depends on the available calves at the Research farm. Yet, female calves had greater plasma concentrations of total protein, glucose, insulin and IGFBPs in the present study, but gender did not affect the growth performance and plasma IGF-I. These findings are surprising because growth rates and plasma IGF-I are typically greater in male than in female calves [36,85–87], but not all studies indicate differences in the growth rate or plasma IGF-I during the milk-fed period [88,89]. One explanation could be that in the present study, the birth weight was not affected by gender, but birth weight is strongly correlated with the subsequent body weight in calves [90]. In a previous studies, the plasma IGFBP-2 concentration was lower in males than in females [86], although no gender effect on the plasma IGFBP-3 concentration was observed [87], in contrast to increased plasma IGFBP-3 in males of several species [86]. Obviously, the gender effects are more distinct when calves are older and sexual steroids are involved in the regulation of the somatotropic axis during puberty [37,86], which was not the case in the present study. The breed effects were less dominant in the present study, because the Holstein breed was also the dominant breed in F3 Holstein x Charolais calves. However, increased plasma concentrations of glucose, NEFA, and triglycerides but lower plasma urea in Holstein calves probably demonstrate potential metabolic changes as a result of different metabolic breeding types [91].

In conclusion, feeding calves with unlimited amounts of MR for the first five weeks of life increases MR intake and the average daily gain but does not impair concentrate intake. The increased body weight and muscle mass at the end of the study mirror the long-lasting anabolic effects reflecting intensive MR feeding and elevated nutrient intake after birth. The metabolic and endocrine changes in blood plasma are most obvious during the intensive MR-feeding period but decrease thereafter. However, increased plasma IGF-I and elevated concentrate intake at the end of the study period indicate enhanced anabolic metabolism resulting from increased solid feed intake in ADLIB calves. MR feeding intensity neither influences the incidence of diarrhea nor affects immunological traits; however, the elevated plasma fibrinogen concentration in RES may imply a feeding effect on the hepatic acute phase protein response. However, whether an intensive milk feeding program for pre-weaning calves helps to avoid diseases can be best investigated in studies with larger calf numbers than in the present study.

## Supporting Information

S1 Fig Cuts indicating the German cutting schema [39]. (PDF)

S1 Table Complete data set of parameters regarding growth and health as shown in Fig 1. (PDF)

S2 Table Complete data set of data after harvest as shown in Table 3. (PDF)

S3 Table Complete data set of plasma concentrations of metabolites and hormones as shown in Figs 2 to 5. (PDF)

S4 Table Complete data set of hepatic mRNA expression. (PDF)
