# Supplementary figures and images for: Effects of Feeding Milk Replacer Ad Libitum or in Restricted Amounts for the First Five Weeks of Life on the Growth, Metabolic Adaptation, and Immune Status of Newborn Calves
Source: PLoS One. 2016 Dec 30;11(12):e0168974. doi: 10.1371/journal.pone.0168974 (PMC5201283; doi:10.1371/journal.pone.0168974)

S1 Fig. Cuts referred to the German cutting schema [39]

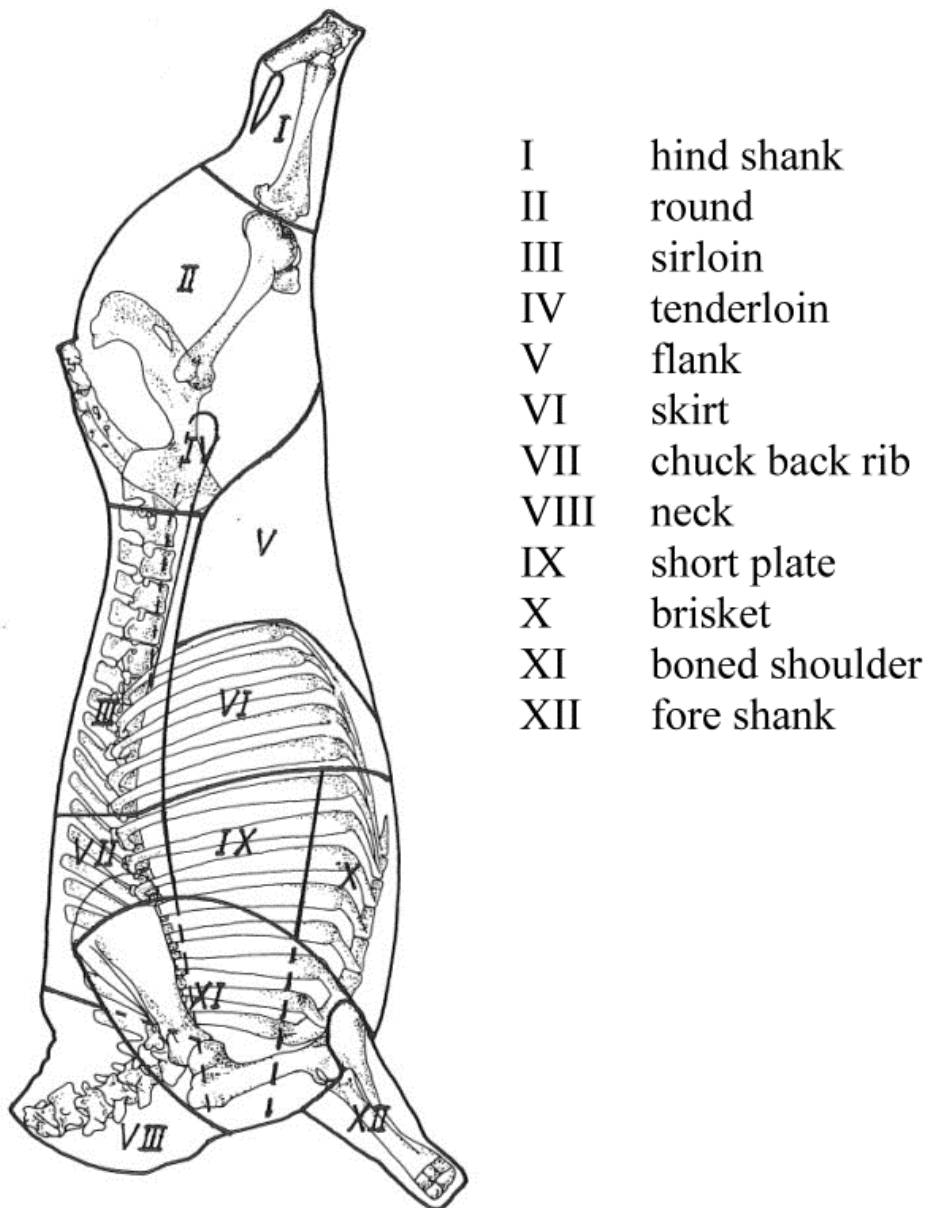

Supplement: S1 Fig — (PDF) [file pone.0168974.s001.pdf]
