# Supplementary material for: Effects of Feeding Milk Replacer Ad Libitum or in Restricted Amounts for the First Five Weeks of Life on the Growth, Metabolic Adaptation, and Immune Status of Newborn Calves
Source: PLoS One. 2016 Dec 30;11(12):e0168974. doi: 10.1371/journal.pone.0168974 (PMC5201283; doi:10.1371/journal.pone.0168974)
Supplement: S1 Table — (PDF) [file pone.0168974.s002.pdf]

S1 Table. Complete data set of parameters regarding growth and health as shown in Fig 1.

Calf	Group	Breed	Sex	Week of life	Milk intake (L)	Concentrate intake (g)	Average daily gain (g)
59857	RES	HF	m	1	5.2	0	643
59858	RES	SEG	w	1	4.9	4	500
59859	RES	SEG	m	1	5.0	0	857
59860	RES	SEG	m	1	4.9	0	500
59861	RES	SEG	w	1	3.0	0	-286
59862	ADL	SEG	m	1	5.7	0	286
59863	ADL	HF	w	1	5.1	0	143
59864	ADL	SEG	m	1	6.3	0	214
59866	ADL	SEG	w	1	4.6	0	143
59867	ADL	SEG	m	1	6.5	0	143
59868	RES	SEG	m	1	4.2	0	143
59869	ADL	SEG	m	1	5.3	0	286
59870	RES	SEG	m	1	4.9	0	71
59871	ADL	HF	m	1	7.4	0	857
59872	RES	SEG	m	1	4.8	0	571
59873	ADL	SEG	m	1	7.9	0	714
59874	RES	SEG	m	1	4.9	0	71
59875	ADL	HF	m	1	5.7	0	214
59876	RES	HF	m	1	4.7	0	214
59877	ADL	SEG	w	1	6.8	0	571
59878	RES	SEG	m	1	5.3	0	143
59879	ADL	SEG	w	1	6.9	0	786
59880	RES	SEG	m	1	5.1	0	429
59881	ADL	SEG	m	1	6.3	0	71
59882	RES	SEG	w	1	4.0	0	71
59883	ADL	SEG	w	1	6.0	0	0
59885	ADL	SEG	w	1	6.6	0	357
59886	RES	SEG	m	1	3.9	0	0
59857	RES	HF	m	2	5.9	9	429
59858	RES	SEG	w	2	5.9	4	143
59859	RES	SEG	m	2	5.7	0	-143
59860	RES	SEG	m	2	6.0	16	71
59861	RES	SEG	w	2	4.5	0	-214
59862	ADL	SEG	m	2	9.9	0	286
59863	ADL	HF	w	2	12.1	0	857
59864	ADL	SEG	m	2	12.9	0	571
59866	ADL	SEG	w	2	6.9	0	71
59867	ADL	SEG	m	2	7.3	24	-71
59868	RES	SEG	m	2	4.9	7	-571
59869	ADL	SEG	m	2	5.5	0	-214

Calf	Group	Breed	Sex	Week of life	Body weight (kg)
59857	RES	HF	m	Birth	52.5
59858	RES	SEG	w	Birth	48.5
59859	RES	SEG	m	Birth	52.0
59860	RES	SEG	m	Birth	38.5
59861	RES	SEG	w	Birth	51.0
59862	ADL	SEG	m	Birth	47.0
59863	ADL	HF	w	Birth	46.0
59864	ADL	SEG	m	Birth	40.0
59866	ADL	SEG	w	Birth	44.0
59867	ADL	SEG	m	Birth	64.0
59868	RES	SEG	m	Birth	49.5
59869	ADL	SEG	m	Birth	42.5
59870	RES	SEG	m	Birth	43.0
59871	ADL	HF	m	Birth	37.0
59872	RES	SEG	m	Birth	52.0
59873	ADL	SEG	m	Birth	46.0
59874	RES	SEG	m	Birth	54.0
59875	ADL	HF	m	Birth	39.0
59876	RES	HF	m	Birth	35.0
59877	ADL	SEG	w	Birth	38.5
59878	RES	SEG	m	Birth	56.0
59879	ADL	SEG	w	Birth	40.5
59880	RES	SEG	m	Birth	45.5
59881	ADL	SEG	m	Birth	54.0
59882	RES	SEG	w	Birth	41.0
59883	ADL	SEG	w	Birth	47.0
59885	ADL	SEG	w	Birth	45.5
59886	RES	SEG	m	Birth	42.0
59857	RES	HF	m	1	57.0
59858	RES	SEG	w	1	52.0
59859	RES	SEG	m	1	58.0
59860	RES	SEG	m	1	42.0
59861	RES	SEG	w	1	49.0
59862	ADL	SEG	m	1	49.0
59863	ADL	HF	w	1	47.0
59864	ADL	SEG	m	1	41.5
59866	ADL	SEG	w	1	45.0
59867	ADL	SEG	m	1	65.0
59868	RES	SEG	m	1	50.5
59869	ADL	SEG	m	1	44.5

Calf	Group	Breed	Sex	Week of life	Milk intake (L)	Concentrate intake (g)	Average daily gain (g)
59870	RES	SEG	m	2	5.9	13	71
59871	ADL	HF	m	2	7.7	0	0
59872	RES	SEG	m	2	5.8	0	-500
59873	ADL	SEG	m	2	9.6	4	286
59874	RES	SEG	m	2	5.6	3	143
59875	ADL	HF	m	2	8.4	0	429
59876	RES	HF	m	2	4.3	0	-71
59877	ADL	SEG	w	2	12.6	59	643
59878	RES	SEG	m	2	4.8	10	-286
59879	ADL	SEG	w	2	9.5	16	286
59880	RES	SEG	m	2	5.0	0	-143
59881	ADL	SEG	m	2	13.3	0	786
59882	RES	SEG	w	2	5.7	0	214
59883	ADL	SEG	w	2	7.1	0	214
59885	ADL	SEG	w	2	11.4	9	429
59886	RES	SEG	m	2	4.5	0	143
59857	RES	HF	m	3	5.9	17	857
59858	RES	SEG	w	3	5.9	13	286
59859	RES	SEG	m	3	5.7	47	286
59860	RES	SEG	m	3	5.7	63	429
59861	RES	SEG	w	3	5.9	20	643
59862	ADL	SEG	m	3	15.0	0	1429
59863	ADL	HF	w	3	16.4	29	1143
59864	ADL	SEG	m	3	11.1	16	643
59866	ADL	SEG	w	3	9.3	21	0
59867	ADL	SEG	m	3	11.4	124	143
59868	RES	SEG	m	3	5.8	99	-143
59869	ADL	SEG	m	3	6.9	0	
59870	RES	SEG	m	3	5.6	0	71
59871	ADL	HF	m	3	13.3	0	1286
59872	RES	SEG	m	3	5.4	24	286
59873	ADL	SEG	m	3	16.3	23	1000
59874	RES	SEG	m	3	5.6	89	286
59875	ADL	HF	m	3	5.5	4	357
59876	RES	HF	m	3	5.6	34	500
59877	ADL	SEG	w	3	15.4	121	1000
59878	RES	SEG	m	3	5.4	86	0
59879	ADL	SEG	w	3	13.4	17	571
59880	RES	SEG	m	3	5.4	166	143
59881	ADL	SEG	m	3	15.4	9	571

Calf	Group	Breed	Sex	Week of life	Body weight (kg)
59870	RES	SEG	m	1	43.5
59871	ADL	HF	m	1	43.0
59872	RES	SEG	m	1	56.0
59873	ADL	SEG	m	1	51.0
59874	RES	SEG	m	1	54.5
59875	ADL	HF	m	1	40.5
59876	RES	HF	m	1	36.5
59877	ADL	SEG	w	1	42.5
59878	RES	SEG	m	1	57.0
59879	ADL	SEG	w	1	46.0
59880	RES	SEG	m	1	48.5
59881	ADL	SEG	m	1	54.5
59882	RES	SEG	w	1	41.5
59883	ADL	SEG	w	1	47.0
59885	ADL	SEG	w	1	48.0
59886	RES	SEG	m	1	42.0
59857	RES	HF	m	2	60.0
59858	RES	SEG	w	2	53.0
59859	RES	SEG	m	2	57.0
59860	RES	SEG	m	2	42.5
59861	RES	SEG	w	2	47.5
59862	ADL	SEG	m	2	51.0
59863	ADL	HF	w	2	53.0
59864	ADL	SEG	m	2	45.5
59866	ADL	SEG	w	2	45.5
59867	ADL	SEG	m	2	64.5
59868	RES	SEG	m	2	46.5
59869	ADL	SEG	m	2	43.0
59870	RES	SEG	m	2	44.0
59871	ADL	HF	m	2	43.0
59872	RES	SEG	m	2	52.5
59873	ADL	SEG	m	2	53.0
59874	RES	SEG	m	2	55.5
59875	ADL	HF	m	2	43.5
59876	RES	HF	m	2	36.0
59877	ADL	SEG	w	2	47.0
59878	RES	SEG	m	2	55.0
59879	ADL	SEG	w	2	48.0
59880	RES	SEG	m	2	47.5
59881	ADL	SEG	m	2	60.0

Calf	Group	Breed	Sex	Week of life	Milk intake (L)	Concentrate intake (g)	Average daily gain (g)
59882	RES	SEG	w	3	5.7	6	429
59883	ADL	SEG	w	3	6.1	11	-286
59885	ADL	SEG	w	3	13.4	49	643
59886	RES	SEG	m	3	5.4	74	0
59857	RES	HF	m	4	5.9	44	429
59858	RES	SEG	w	4	5.9	106	500
59859	RES	SEG	m	4	5.7	20	429
59860	RES	SEG	m	4	6.0	37	500
59861	RES	SEG	w	4	5.9	0	286
59862	ADL	SEG	m	4	15.1	9	1071
59863	ADL	HF	w	4	14.6	21	429
59864	ADL	SEG	m	4	14.0	50	929
59866	ADL	SEG	w	4	12.7	21	857
59867	ADL	SEG	m	4	10.9	96	786
59868	RES	SEG	m	4	5.6	221	571
59869	ADL	SEG	m	4	9.9	11	464
59870	RES	SEG	m	4	5.6	10	500
59871	ADL	HF	m	4	16.3	0	786
59872	RES	SEG	m	4	5.9	89	429
59873	ADL	SEG	m	4	12.8	41	857
59874	RES	SEG	m	4	5.4	133	571
59875	ADL	HF	m	4	10.4	0	857
59876	RES	HF	m	4	5.8	0	786
59877	ADL	SEG	w	4	16.9	110	1286
59878	RES	SEG	m	4	5.4	121	357
59879	ADL	SEG	w	4	13.7	54	1429
59880	RES	SEG	m	4	5.7	199	500
59881	ADL	SEG	m	4	14.3	74	1214
59882	RES	SEG	w	4	5.8	141	214
59883	ADL	SEG	w	4	13.6	149	1071
59885	ADL	SEG	w	4	16.3	44	857
59886	RES	SEG	m	4	5.5	69	714
59857	RES	HF	m	5	5.9	50	214
59858	RES	SEG	w	5	5.7	321	1357
59859	RES	SEG	m	5	5.9	0	357
59860	RES	SEG	m	5	5.8	349	643
59861	RES	SEG	w	5	5.9	136	643
59862	ADL	SEG	m	5	10.9	66	0
59863	ADL	HF	w	5	17.9	230	857
59864	ADL	SEG	m	5	14.9	146	1000

Calf	Group	Breed	Sex	Week of life	Body weight (kg)
59882	RES	SEG	w	2	43.0
59883	ADL	SEG	w	2	48.5
59885	ADL	SEG	w	2	51.0
59886	RES	SEG	m	2	43.0
59857	RES	HF	m	3	66.0
59858	RES	SEG	w	3	55.0
59859	RES	SEG	m	3	59.0
59860	RES	SEG	m	3	45.5
59861	RES	SEG	w	3	52.0
59862	ADL	SEG	m	3	61.0
59863	ADL	HF	w	3	61.0
59864	ADL	SEG	m	3	50.0
59866	ADL	SEG	w	3	45.5
59867	ADL	SEG	m	3	65.5
59868	RES	SEG	m	3	45.5
59869	ADL	SEG	m	3	
59870	RES	SEG	m	3	44.5
59871	ADL	HF	m	3	52.0
59872	RES	SEG	m	3	54.5
59873	ADL	SEG	m	3	60.0
59874	RES	SEG	m	3	57.5
59875	ADL	HF	m	3	46.0
59876	RES	HF	m	3	39.5
59877	ADL	SEG	w	3	54.0
59878	RES	SEG	m	3	55.0
59879	ADL	SEG	w	3	52.0
59880	RES	SEG	m	3	48.5
59881	ADL	SEG	m	3	64.0
59882	RES	SEG	w	3	46.0
59883	ADL	SEG	w	3	46.5
59885	ADL	SEG	w	3	55.5
59886	RES	SEG	m	3	43.0
59857	RES	HF	m	4	69.0
59858	RES	SEG	w	4	58.5
59859	RES	SEG	m	4	62.0
59860	RES	SEG	m	4	49.0
59861	RES	SEG	w	4	54.0
59862	ADL	SEG	m	4	68.5
59863	ADL	HF	w	4	64.0
59864	ADL	SEG	m	4	56.5

Calf	Group	Breed	Sex	Week of life	Milk intake (L)	Concentrate intake (g)	Average daily gain (g)
59866	ADL	SEG	w	5	13.8	69	1214
59867	ADL	SEG	m	5	10.6	324	714
59868	RES	SEG	m	5	6.0	346	571
59869	ADL	SEG	m	5	14.9	9	1071
59870	RES	SEG	m	5	5.9	64	500
59871	ADL	HF	m	5	11.4	0	786
59872	RES	SEG	m	5	5.9	197	786
59873	ADL	SEG	m	5	12.9	187	429
59874	RES	SEG	m	5	5.5	191	429
59875	ADL	HF	m	5	14.9	33	643
59876	RES	HF	m	5	5.8	0	571
59877	ADL	SEG	w	5	16.6	130	1000
59878	RES	SEG	m	5	5.4	284	929
59879	ADL	SEG	w	5	16.2	0	857
59880	RES	SEG	m	5	5.6	686	786
59881	ADL	SEG	m	5	14.1	60	500
59882	RES	SEG	w	5	5.9	186	714
59883	ADL	SEG	w	5	16.6	90	714
59885	ADL	SEG	w	5	17.4	20	1071
59886	RES	SEG	m	5	5.6	189	143
59857	RES	HF	m	6	5.8	121	857
59858	RES	SEG	w	6	5.9	317	214
59859	RES	SEG	m	6	5.9	357	643
59860	RES	SEG	m	6	5.6	547	643
59861	RES	SEG	w	6	4.9	213	214
59862	ADL	SEG	m	6	9.2	503	500
59863	ADL	HF	w	6	11.1	341	929
59864	ADL	SEG	m	6	9.4	463	857
59866	ADL	SEG	w	6	12.3	121	786
59867	ADL	SEG	m	6	8.1	461	1000
59868	RES	SEG	m	6	5.9	360	643
59869	ADL	SEG	m	6	9.3	54	500
59870	RES	SEG	m	6	5.8	116	857
59871	ADL	HF	m	6	9.7	103	143
59872	RES	SEG	m	6	5.7	516	643
59873	ADL	SEG	m	6	9.4	306	786
59874	RES	SEG	m	6	5.4	107	286
59875	ADL	HF	m	6	10.1	134	571
59876	RES	HF	m	6	5.8	150	429
59877	ADL	SEG	w	6	11.4	359	571

Calf	Group	Breed	Sex	Week of life	Body weight (kg)
59866	ADL	SEG	w	4	51.5
59867	ADL	SEG	m	4	71.0
59868	RES	SEG	m	4	49.5
59869	ADL	SEG	m	4	49.5
59870	RES	SEG	m	4	48.0
59871	ADL	HF	m	4	57.5
59872	RES	SEG	m	4	57.5
59873	ADL	SEG	m	4	66.0
59874	RES	SEG	m	4	61.5
59875	ADL	HF	m	4	52.0
59876	RES	HF	m	4	45.0
59877	ADL	SEG	w	4	63.0
59878	RES	SEG	m	4	57.5
59879	ADL	SEG	w	4	62.0
59880	RES	SEG	m	4	52.0
59881	ADL	SEG	m	4	72.5
59882	RES	SEG	w	4	47.5
59883	ADL	SEG	w	4	54.0
59885	ADL	SEG	w	4	61.5
59886	RES	SEG	m	4	48.0
59857	RES	HF	m	5	70.5
59858	RES	SEG	w	5	68.0
59859	RES	SEG	m	5	64.5
59860	RES	SEG	m	5	53.5
59861	RES	SEG	w	5	58.5
59862	ADL	SEG	m	5	68.5
59863	ADL	HF	w	5	70.0
59864	ADL	SEG	m	5	63.5
59866	ADL	SEG	w	5	60.0
59867	ADL	SEG	m	5	76.0
59868	RES	SEG	m	5	53.5
59869	ADL	SEG	m	5	57.0
59870	RES	SEG	m	5	51.5
59871	ADL	HF	m	5	63.0
59872	RES	SEG	m	5	63.0
59873	ADL	SEG	m	5	69.0
59874	RES	SEG	m	5	64.5
59875	ADL	HF	m	5	56.5
59876	RES	HF	m	5	49.0
59877	ADL	SEG	w	5	70.0

Calf	Group	Breed	Sex	Week of life	Milk intake (L)	Concentrate intake (g)	Average daily gain (g)
59878	RES	SEG	m	6	5.1	590	571
59879	ADL	SEG	w	6	9.4	176	429
59880	RES	SEG	m	6	5.4	904	1143
59881	ADL	SEG	m	6	9.0	544	429
59882	RES	SEG	w	6	5.9	301	429
59883	ADL	SEG	w	6	9.4	477	786
59885	ADL	SEG	w	6	9.6	441	357
59886	RES	SEG	m	6	5.4	486	357
59857	RES	HF	m	7	5.7	356	857
59858	RES	SEG	w	7	5.9	1250	929
59859	RES	SEG	m	7	5.7	1009	1143
59860	RES	SEG	m	7	5.6	709	286
59861	RES	SEG	w	7	5.6	716	857
59862	ADL	SEG	m	7	6.1	574	1000
59863	ADL	HF	w	7	6.1	497	429
59864	ADL	SEG	m	7	6.0	643	857
59866	ADL	SEG	w	7	6.1	369	786
59867	ADL	SEG	m	7	6.2	1130	714
59868	RES	SEG	m	7	5.9	359	1143
59869	ADL	SEG	m	7	6.0	20	500
59870	RES	SEG	m	7	5.6	224	929
59871	ADL	HF	m	7	6.1	376	786
59872	RES	SEG	m	7	5.8	1144	714
59873	ADL	SEG	m	7	5.9	441	357
59874	RES	SEG	m	7	5.4	149	714
59875	ADL	HF	m	7	6.0	253	857
59876	RES	HF	m	7	5.6	323	714
59877	ADL	SEG	w	7	6.0	711	500
59878	RES	SEG	m	7	5.2	520	786
59879	ADL	SEG	w	7	6.1	521	714
59880	RES	SEG	m	7	5.9	1603	929
59881	ADL	SEG	m	7	6.1	997	1071
59882	RES	SEG	w	7	5.7	973	643
59883	ADL	SEG	w	7	5.9	1009	643
59885	ADL	SEG	w	7	5.9	756	643
59886	RES	SEG	m	7	5.4	639	786
59857	RES	HF	m	8	5.8	949	929
59858	RES	SEG	w	8	5.6	829	857
59859	RES	SEG	m	8	5.1	764	229
59860	RES	SEG	m	8	5.4	1674	1071

Calf	Group	Breed	Sex	Week of life	Body weight (kg)
59878	RES	SEG	m	5	64.0
59879	ADL	SEG	w	5	68.0
59880	RES	SEG	m	5	57.5
59881	ADL	SEG	m	5	76.0
59882	RES	SEG	w	5	52.5
59883	ADL	SEG	w	5	59.0
59885	ADL	SEG	w	5	69.0
59886	RES	SEG	m	5	49.0
59857	RES	HF	m	6	76.5
59858	RES	SEG	w	6	69.5
59859	RES	SEG	m	6	69.0
59860	RES	SEG	m	6	58.0
59861	RES	SEG	w	6	60.0
59862	ADL	SEG	m	6	72.0
59863	ADL	HF	w	6	76.5
59864	ADL	SEG	m	6	69.5
59866	ADL	SEG	w	6	65.5
59867	ADL	SEG	m	6	83.0
59868	RES	SEG	m	6	58.0
59869	ADL	SEG	m	6	60.5
59870	RES	SEG	m	6	57.5
59871	ADL	HF	m	6	64.0
59872	RES	SEG	m	6	67.5
59873	ADL	SEG	m	6	74.5
59874	RES	SEG	m	6	66.5
59875	ADL	HF	m	6	60.5
59876	RES	HF	m	6	52.0
59877	ADL	SEG	w	6	74.0
59878	RES	SEG	m	6	68.0
59879	ADL	SEG	w	6	71.0
59880	RES	SEG	m	6	65.5
59881	ADL	SEG	m	6	79.0
59882	RES	SEG	w	6	55.5
59883	ADL	SEG	w	6	64.5
59885	ADL	SEG	w	6	71.5
59886	RES	SEG	m	6	51.5
59857	RES	HF	m	7	82.5
59858	RES	SEG	w	7	76.0
59859	RES	SEG	m	7	77.0
59860	RES	SEG	m	7	60.0

Calf	Group	Breed	Sex	Week of life	Milk intake (L)	Concentrate intake (g)	Average daily gain (g)
59861	RES	SEG	w	8	5.4	1404	1214
59862	ADL	SEG	m	8	6.0	1097	929
59863	ADL	HF	w	8	5.9	1214	1071
59864	ADL	SEG	m	8	6.1	1157	1000
59866	ADL	SEG	w	8	6.0	690	643
59867	ADL	SEG	m	8	5.4	2004	1071
59868	RES	SEG	m	8	5.8	641	571
59869	ADL	SEG	m	8	6.0	224	857
59870	RES	SEG	m	8	5.7	284	714
59871	ADL	HF	m	8	6.0	520	1000
59872	RES	SEG	m	8	5.7	1101	786
59873	ADL	SEG	m	8	6.0	499	429
59874	RES	SEG	m	8	5.6	334	357
59875	ADL	HF	m	8	6.0	681	929
59876	RES	HF	m	8	5.5	550	571
59877	ADL	SEG	w	8	6.0	1301	786
59878	RES	SEG	m	8	5.6	1129	1000
59879	ADL	SEG	w	8	5.9	1503	857
59880	RES	SEG	m	8	5.1	1890	643
59881	ADL	SEG	m	8	5.9	1204	643
59882	RES	SEG	w	8	5.6	743	286
59883	ADL	SEG	w	8	6.0	2419	714
59885	ADL	SEG	w	8	6.0	994	714
59886	RES	SEG	m	8	4.7	513	714

Calf	Group	Breed	Sex	Week of life	Body weight (kg)
59861	RES	SEG	w	7	66.0
59862	ADL	SEG	m	7	79.0
59863	ADL	HF	w	7	79.5
59864	ADL	SEG	m	7	75.5
59866	ADL	SEG	w	7	71.0
59867	ADL	SEG	m	7	88.0
59868	RES	SEG	m	7	66.0
59869	ADL	SEG	m	7	64.0
59870	RES	SEG	m	7	64.0
59871	ADL	HF	m	7	69.5
59872	RES	SEG	m	7	72.5
59873	ADL	SEG	m	7	77.0
59874	RES	SEG	m	7	71.5
59875	ADL	HF	m	7	66.5
59876	RES	HF	m	7	57.0
59877	ADL	SEG	w	7	77.5
59878	RES	SEG	m	7	73.5
59879	ADL	SEG	w	7	76.0
59880	RES	SEG	m	7	72.0
59881	ADL	SEG	m	7	86.5
59882	RES	SEG	w	7	60.0
59883	ADL	SEG	w	7	69.0
59885	ADL	SEG	w	7	76.0
59886	RES	SEG	m	7	57.0
59857	RES	HF	m	8	89.0
59858	RES	SEG	w	8	82.0
59859	RES	SEG	m	8	78.6
59860	RES	SEG	m	8	67.5
59861	RES	SEG	w	8	74.5
59862	ADL	SEG	m	8	85.5
59863	ADL	HF	w	8	87.0
59864	ADL	SEG	m	8	82.5
59866	ADL	SEG	w	8	75.5
59867	ADL	SEG	m	8	95.5
59868	RES	SEG	m	8	70.0
59869	ADL	SEG	m	8	70.0
59870	RES	SEG	m	8	69.0
59871	ADL	HF	m	8	76.5
59872	RES	SEG	m	8	78.0
59873	ADL	SEG	m	8	80.0

Calf	Group	Breed	Sex	Week of life	Milk intake (L)	Concentrate intake (g)	Average daily gain (g)

Calf	Group	Breed	Sex	Week of life	Body weight (kg)
59874	RES	SEG	m	8	74.0
59875	ADL	HF	m	8	73.0
59876	RES	HF	m	8	61.0
59877	ADL	SEG	w	8	83.0
59878	RES	SEG	m	8	80.5
59879	ADL	SEG	w	8	82.0
59880	RES	SEG	m	8	76.5
59881	ADL	SEG	m	8	91.0
59882	RES	SEG	w	8	62.0
59883	ADL	SEG	w	8	74.0
59885	ADL	SEG	w	8	81.0
59886	RES	SEG	m	8	62.0

Calf	Group	Breed	Sex	Days treated because of scours
59857	RES	HF	m	6
59858	RES	SEG	w	7
59859	RES	SEG	m	0
59860	RES	SEG	m	3
59861	RES	SEG	w	3
59862	ADL	SEG	m	5
59863	ADL	HF	w	0
59864	ADL	SEG	m	8
59866	ADL	SEG	w	14
59867	ADL	SEG	m	6
59868	RES	SEG	m	0
59869	ADL	SEG	m	3
59870	RES	SEG	m	0
59871	ADL	HF	m	9
59872	RES	SEG	m	4
59873	ADL	SEG	m	4
59874	RES	SEG	m	3
59875	ADL	HF	m	0
59876	RES	HF	m	0
59877	ADL	SEG	w	0
59878	RES	SEG	m	0
59879	ADL	SEG	w	0
59880	RES	SEG	m	0
59881	ADL	SEG	m	0
59882	RES	SEG	w	0
59883	ADL	SEG	w	3
59885	ADL	SEG	w	0
59886	RES	SEG	m	0
