# Supplementary material for: Effects of Feeding Milk Replacer Ad Libitum or in Restricted Amounts for the First Five Weeks of Life on the Growth, Metabolic Adaptation, and Immune Status of Newborn Calves
Source: PLoS One. 2016 Dec 30;11(12):e0168974. doi: 10.1371/journal.pone.0168974 (PMC5201283; doi:10.1371/journal.pone.0168974)
Supplement: S2 Table — (PDF) [file pone.0168974.s003.pdf]

S2 Table. Complete data set of data after harvest as shown in Table 3.

Calf	Group	Breed	Sex	Hot carcass weight, left side (kg)	Liver (kg)	Pancreas (kg)	Perirenal fat (kg)	Hind shank (kg)	Round (kg)	Sirloin (kg)	Tenderloin (kg)	Flank (kg)	Skirt (kg)	Chuck back rib (kg)
59857	RES	HF	m	23.4	1.45	0.09	0.35	0.65	5.52	0.99	0.50	0.65	0.34	1.23
59858	RES	SEG	w	22.0	1.41	0.11	0.25	0.72	5.30	1.01	0.47	0.66	0.29	1.21
59859	RES	SEG	m	23.4	1.55	0.13	0.17	0.86	5.47	1.01	0.48	0.61	0.36	1.21
59860	RES	SEG	m	18.8	1.23	0.07	0.22	0.63	4.43	0.88	0.41	0.58	0.29	1.02
59861	RES	SEG	w	19.8	1.49	0.09	0.22	0.62	4.64	0.80	0.45	0.47	0.43	1.07
59862	ADL	SEG	m	24.0	1.66	0.07	0.53	0.82	5.61	1.27	0.51	0.80	0.41	1.35
59863	ADL	HF	w	23.2	1.45	0.08	0.74	0.67	5.68	1.09	0.51	0.77	0.46	1.23
59864	ADL	SEG	m	22.0	1.30	0.07	0.48	0.75	5.09	1.02	0.46	0.53	0.57	1.32
59866	ADL	SEG	w	21.2	1.44	0.07	0.50	0.75	5.30	1.00	0.44	0.63	0.43	1.21
59867	ADL	SEG	m	27.0	2.04	0.11	0.20	0.92	6.44	1.12	0.56	0.82	0.38	1.43
59868	RES	SEG	m	19.5	1.07	0.07	0.22	0.65	4.82	0.87	0.35	0.59	0.37	1.07
59869	ADL	SEG	m	18.9	1.21	0.09	0.29	0.61	4.49	0.89	0.38	0.54	0.40	1.01
59870	RES	SEG	m	20.0	1.64	0.07	0.32	0.61	4.72	0.88	0.37	0.52	0.30	1.15
59871	ADL	HF	m	21.4	1.52	0.09	0.36	0.74	5.28	1.09	0.41	0.58	0.41	1.23
59872	RES	SEG	m	21.5	1.46	0.07	0.20	0.69	4.86	0.89	0.40	0.64	0.41	1.10
59873	ADL	SEG	m	23.0	1.49	0.07	0.41	0.72	5.46	1.02	0.50	0.63	0.41	1.11
59874	RES	SEG	m	21.3	1.21	0.05	0.16	0.68	4.87	0.85	0.38	0.57	0.40	1.07
59875	ADL	HF	m	19.2	1.40	0.07	0.31	0.62	4.57	0.79	0.37	0.60	0.31	0.96
59876	RES	HF	m	16.2	1.38	0.07	0.32	0.51	3.66	1.15	0.30	0.48	0.32	0.77
59877	ADL	SEG	w	23.6	1.67	0.11	0.82	0.82	5.70	1.09	0.53	0.80	0.51	1.29
59878	RES	SEG	m	21.2	1.51	0.09	0.31	0.69	4.46	0.82	0.38	0.70	0.38	1.16
59879	ADL	SEG	w	23.2	1.39	0.08	0.39	0.89	5.87	1.12	0.55	0.70	0.54	1.43
59880	RES	SEG	m	20.2	1.49	0.05	0.34	0.69	4.71	0.79	0.39	0.58	0.34	1.01
59881	ADL	SEG	m	25.5	1.65	0.10	0.41	0.91	6.65	1.17	0.52	0.73	0.43	1.35
59882	RES	SEG	w	19.9	1.26	0.08	0.34	0.64	5.15	0.95	0.42	0.53	0.36	1.17
59883	ADL	SEG	w	19.4	1.34	0.09	0.44	0.63	4.38	0.77	0.41	0.60	0.32	0.72
59885	ADL	SEG	w	22.3	1.47	0.07	0.38	0.73	5.46	1.05	0.49	0.59	0.45	1.29
59886	RES	SEG	m	16.2	1.20	0.07	0.34	0.57	3.79	0.62	0.29	0.52	0.31	0.90

Calf	Group	Breed	Sex	Neck (kg)	Short plate (kg)	Brisket (kg)	Boned shoulder (kg)	Fore shank (kg)	Sum of muscle (kg)	Sum of subcutaneous fat (kg)	MLD length (cm)	MLD circumference (cm)
59857	RES	HF	m	1.68	0.74	0.43	2.08	0.43	13.15	0.11	52	21
59858	RES	SEG	w	1.34	0.54	0.59	2.08	0.43	12.57	0.00	50	20
59859	RES	SEG	m	1.59	0.48	0.76	2.20	0.51	13.37	0.06	51	19
59860	RES	SEG	m	1.21	0.57	0.49	1.75	0.41	10.91	0.00	47	19
59861	RES	SEG	w	1.16	0.54	0.45	1.92	0.39	11.02	0.01	47	19
59862	ADL	SEG	m	1.57	0.66	0.77	2.24	0.54	14.31	0.00	50	21
59863	ADL	HF	w	1.52	0.67	0.74	2.22	0.46	13.82	0.17	51	21
59864	ADL	SEG	m	1.25	0.78	0.86	2.00	0.48	12.44	0.12	50	20
59866	ADL	SEG	w	1.03	0.66	0.87	1.94	0.46	12.76	0.07	51	21
59867	ADL	SEG	m	1.78	0.66	1.09	2.68	0.62	15.83	0.06	53	21
59868	RES	SEG	m	1.00	0.69	0.52	1.90	0.37	11.31	0.04	49	20
59869	ADL	SEG	m	1.24	0.55	0.55	1.67	0.41	11.07	0.01	46	19
59870	RES	SEG	m	1.06	0.65	0.72	1.86	0.40	11.37	0.02	47	19
59871	ADL	HF	m	1.09	0.65	0.63	1.83	0.46	12.56	0.01	54	20
59872	RES	SEG	m	1.24	0.66	0.56	1.88	0.42	11.84	0.00	50	18
59873	ADL	SEG	m	1.63	0.65	0.96	2.12	0.46	13.55	0.01	51	21
59874	RES	SEG	m	1.28	0.76	0.79	1.86	0.41	12.05	0.00	49	18
59875	ADL	HF	m	1.22	0.49	0.54	1.69	0.42	10.88	0.00	45	17
59876	RES	HF	m	0.83	0.44	0.37	1.38	0.34	9.17	0.00	45	17
59877	ADL	SEG	w	1.48	0.62	0.82	2.11	0.50	14.15	0.01	48	19
59878	RES	SEG	m	1.24	0.54	0.63	1.94	0.50	11.49	0.00	46	17
59879	ADL	SEG	w	1.53	0.68	0.67	2.21	0.47	14.44	0.00	51	20
59880	RES	SEG	m	1.28	0.49	0.61	1.80	0.42	11.32	0.00	46	17
59881	ADL	SEG	m	1.81	0.64	0.94	2.34	0.54	15.69	0.00	48	20
59882	RES	SEG	w	1.28	0.57	0.62	1.74	0.40	12.08	0.00	46	20
59883	ADL	SEG	w	1.24	0.64	0.60	1.84	0.41	10.73	0.00	47	17
59885	ADL	SEG	w	1.60	0.54	0.85	1.97	0.46	13.51	0.05	55	19
59886	RES	SEG	m	1.12	0.42	0.52	1.52	0.37	9.44	0.01	45	16
