# Supplementary material for: Effects of Feeding Milk Replacer Ad Libitum or in Restricted Amounts for the First Five Weeks of Life on the Growth, Metabolic Adaptation, and Immune Status of Newborn Calves
Source: PLoS One. 2016 Dec 30;11(12):e0168974. doi: 10.1371/journal.pone.0168974 (PMC5201283; doi:10.1371/journal.pone.0168974)
Supplement: S3 Table — (PDF) [file pone.0168974.s004.pdf]

S3 Table. Complete data set of plasma concentrations of metabolites and hormones as shown in Fig 2 to 5.

Calf	Group	Breed	Sex	Day of life	Glucose (mmol/L)	Lactate (mmol/L)	BHB (mmol/L)	NEFA (mmol/L)	Triglycerides (mmol/L)	Cholesterol (mmol/L)	Insulin (uU/mL)	IGF-I (µg/L)	IGFBP3 (µg/L)	IGFBP2 (µg/L)	IGFBP4 (µg/L)	Total protein (g/L)	IgG1 (g/L)	IgG2 (g/L)	IgM (g/L)	Albumin (g/L)	Urea (mmol/L)	Fibrinoge (g/L)	Haptoglobin (g/L)
59857	RES	HF	m	1	3.09	1.29	0.07	1.36	0.29	0.78	12.97	221.91	4.89	0.60	1.77	41.10	0.01	0.01	0.03	23.20	3.21		0.00
59858	RES	SEG	w	1	5.69	4.77	0.02	0.85	0.22	0.65	130.33	182.71	11.82	0.91	3.41	43.10	0.00			24.20	4.79		0.00
59859	RES	SEG	m	1	5.94	7.15	0.02	0.22	0.17	0.48	16.65	197.90	5.94	0.74	2.22	38.20	0.04	0.05	0.03	22.00	5.18	1.00	0.00
59860	RES	SEG	m	1	3.10	2.53	0.04	0.92	0.18	0.55	4.74	84.84	7.26	0.49	2.09	39.40	0.01	0.00	0.02	20.60	4.64	2.83	0.00
59861	RES	SEG	w	1	3.50	1.46	0.01	1.15	0.23	0.41	98.83	192.44	8.32	0.70	2.35	40.80	0.23	0.06	0.50	22.40	2.30	0.63	0.00
59862	ADL	SEG	m	1	4.86	6.12	0.01	0.05	0.09	0.44	6.79	217.59	4.70	0.45	1.73	41.20	0.11	0.02	0.20	23.60	3.70	0.42	0.00
59863	ADL	HF	w	1	5.38	1.59	0.02	0.73	0.14	0.53	21.03	178.52	9.32	1.25	2.83	40.40	0.06		0.05	22.20	5.00	0.58	0.00
59864	ADL	SEG	m	1	4.94	5.58	0.06	0.59	0.15	0.52	27.93	148.79	3.57	0.10	1.40	40.70	0.01	0.06	0.03	23.90	5.12		0.00
59866	ADL	SEG	w	1	5.06	8.55	0.04	0.08	0.09	0.53	12.89	171.58	8.59	0.57	2.48	40.40	0.00	0.04		22.50	4.46	0.89	0.00
59867	ADL	SEG	m	1	9.78	0.05	0.04	0.14	0.36	0.48	13.04	245.65	7.20	0.79	1.95	41.40	0.01	0.00	0.02	22.40	5.90	0.61	0.00
59868	RES	SEG	m	1	2.85	3.79	0.03	0.50	0.16	0.51	37.43	183.06	4.51	0.94	5.59	43.80	0.00	0.00	0.12	24.60	5.74	1.13	0.00
59869	ADL	SEG	m	1	5.31	5.99	0.07	0.07	0.07	0.44	10.52	187.97	2.39	0.10	2.40	37.20	0.01	0.05	0.12	22.50	8.50	0.40	0.00
59870	RES	SEG	m	1	3.90	5.24	0.02	0.03	0.10	0.43	11.50	137.39	6.14	1.58	3.45	45.10	0.01	0.06	0.02	25.00	7.24	0.06	0.00
59871	ADL	HF	m	1	9.99	2.39	0.02	0.62	0.27	0.78			4.14	0.57	1.83	45.10	0.02	0.01	0.11	24.60	2.41	2.41	0.00
59872	RES	SEG	m	1	3.79	3.77	0.02	0.87	0.24	0.44	8.40	225.63	9.04	0.10	1.50	42.90	0.01	0.00	0.03	23.30	4.17	3.06	0.00
59873	ADL	SEG	m	1	3.70	5.53	0.02	0.37	0.16	0.37	24.63	233.56	9.06	0.10	1.07	40.30	0.02	0.02	0.08	23.10	3.76	0.00	0.00
59874	RES	SEG	m	1	0.02	2.40	0.01	0.02	0.16	0.47	8.08	164.24	1.54	0.10	0.75	37.30	0.01	0.00	0.03	20.80	3.22		0.00
59875	ADL	HF	m	1	4.17	1.99	0.01	0.63	0.22	0.39	16.60	78.50	2.03	0.74	1.84	37.30	0.01	0.00	0.02	20.80	4.65		0.00
59876	RES	HF	m	1	2.85	1.52	0.03	0.47	0.16	0.45	5.89	88.43	3.17	0.59	1.46	41.60	0.01	0.00	0.02	23.10	4.65	0.58	0.00
59877	ADL	SEG	w	1	6.76	8.88	0.05	0.37	0.25	0.73	21.45	42.27	3.75	0.91	2.19	59.40	0.04	0.16	0.48	21.80	6.07		0.00
59878	RES	SEG	m	1	3.58	2.70	0.01	0.43	0.15	0.47	13.66	136.34	4.22	0.10	0.57	38.10	0.01	0.00	0.08	21.80	3.97	1.20	0.00
59879	ADL	SEG	w	1	4.98	5.06	0.01	0.30	0.11	0.43	21.50	151.22	5.07	0.10	1.43	48.50	0.01	0.00	0.04	24.90	5.34	3.70	0.00
59880	RES	SEG	m	1	3.91	12.37	0.02	0.11	0.14	0.48	14.65	128.52	4.48	0.58	3.18	44.20	0.00	0.01	0.02	24.20	5.38		0.00
59881	ADL	SEG	m	1	5.94	9.69	0.01	0.22	0.18	0.42	8.15	201.84	6.33	0.10	1.23	42.00	0.01	0.05	0.06	21.70	4.79	4.90	0.00
59882	RES	SEG	w	1	7.10	10.15	0.01	0.23	0.47	0.54	1.92	177.42	7.60	1.29	3.89	38.30	0.02	0.02	0.02	20.60	5.80	0.96	0.00
59883	ADL	SEG	w	1	2.17	4.05	0.01	0.44	0.12	0.62	8.34	169.36	9.89	1.24	3.01	43.50	0.06	0.00	0.02	23.90	3.37		0.00
59885	ADL	SEG	w	1	4.45	3.94	0.03	0.05	0.10	0.32	22.81	158.54	3.63	0.10	1.57	38.10	0.01	0.00	0.02	21.20	3.91		0.00
59886	RES	SEG	m	1	2.74	4.03	0.01	0.50	0.20	0.53	9.97	122.71	5.93	0.75	2.31	42.30	0.01	0.04	0.05	22.50	3.40	1.75	0.00
59857	RES	HF	m	2	7.99	1.46	0.06	0.10	0.14	0.93	45.48	259.62	9.66	1.26	5.44	70.30	21.88	0.19	1.00	19.90	3.67	0.18	0.00
59858	RES	SEG	w	2	6.56	1.55	0.01	0.15	0.25	1.03	60.40	189.95	16.29	2.25	9.56	58.10	16.28	0.13	0.39	21.70	3.60	2.88	0.00
59859	RES	SEG	m	2	8.25	2.82	0.01	0.11	0.16	0.67	142.72	179.53	4.58	0.10	2.07	56.30	6.66	0.28	0.04	20.10	3.98	2.94	0.00
59860	RES	SEG	m	2	5.74	1.56	0.03	0.17	0.09	0.78	7.16	142.64	21.22	2.45	10.48	64.40	15.75	0.50	0.29	18.60	3.32	1.96	0.00
59861	RES	SEG	w	2	5.43	1.63	0.05	0.31	0.21	0.72	15.24	248.57	10.68	0.84	5.76	56.80	16.28	0.50	0.33	19.40	3.74	2.83	0.00
59862	ADL	SEG	m	2	5.66	1.50	0.01	0.39	0.53	0.78	15.02	229.98	6.00	0.43	2.83	54.30	7.06	0.23	0.12	22.30	2.53	3.09	0.00
59863	ADL	HF	w	2	6.36	2.13	0.02	0.46	0.24	0.70	11.49	192.26	8.32	0.93	2.81	48.70	7.21	0.32	0.38	20.90	3.53	3.00	0.00
59864	ADL	SEG	m	2	6.14	4.23	0.04	0.28	0.23	0.70	39.59	161.55	4.75	0.83	3.89	59.10	11.38	0.48	0.35	21.00	4.10	0.20	0.00
59866	ADL	SEG	w	2	6.28	2.92	0.01	0.30	0.35	0.92	33.75	163.13	11.68	0.66	3.63	48.00	10.47	0.45		21.30	4.57	3.21	0.00
59867	ADL	SEG	m	2	5.98	0.05	0.02	0.29	0.21	0.80	35.31	291.40	17.31	1.19	5.45	54.80	14.08	0.29	0.38	21.50	4.56	4.25	0.00
59868	RES	SEG	m	2	4.60	4.87	0.03	0.48	0.35	0.61	30.69	158.28	8.18	1.50	10.71	45.50	14.14	0.26	0.60	23.00	4.82	0.92	0.00
59869	ADL	SEG	m	2	5.59	4.29	0.04	0.23	0.12	0.47	56.76	170.89	5.93	0.75	7.37	56.40	17.01	0.19	0.23	20.30	6.47	0.40	0.00
59870	RES	SEG	m	2	5.65	4.08	0.00	0.26	0.20	0.71	20.30	148.10	11.94	1.35	8.03	56.00	9.03	0.26	0.39	24.00	5.09	0.06	0.00
59871	ADL	HF	m	2	5.69	1.48	0.01	0.36	0.22	0.92	40.38	124.34	13.13	1.59	5.89	59.40	18.43	0.34	0.50	22.90	4.19	1.85	0.00
59872	RES	SEG	m	2	5.04	2.09	0.02	0.19	0.13	0.50	34.76	237.26	10.61	0.10	4.23	63.60	14.50	0.50	0.49	19.70	4.44	3.85	0.00
59873	ADL	SEG	m	2	3.51	3.95	0.01	0.21	0.10	0.33	45.92	216.90	12.11	0.66	2.78	54.80	17.18	0.38	0.38	19.50	2.65	0.34	0.00
59874	RES	SEG	m	2	3.76	2.32	0.02	0.31	0.15	0.47	7.66	151.70	2.89	0.10	1.29	54.90	6.46	0.40	1.60	18.00	3.22		0.00
59875	ADL	HF	m	2	5.85	1.80	0.04	0.49	0.29	0.68	6.47	97.84	3.18	0.72	3.03	59.40	17.18	0.47	0.32	19.30	3.55		0.00
59876	RES	HF	m	2	5.69	2.26	0.04	0.38	0.70	0.66	11.65	109.09	3.39	0.10	2.72	64.30	17.44	0.43	1.50	20.80	3.48	2.45	0.00
59877	ADL	SEG	w	2	8.48	1.90	0.08	0.35	0.23	0.99	10.54	100.69	3.17	0.10	1.98	76.50	12.61	0.50	0.78	21.40	4.93	3.48	0.00
59878	RES	SEG	m	2	5.48	3.08	0.03	0.33	0.23	0.79	10.05	189.10	7.34	0.10	1.61	66.70	21.36	0.24	0.20	20.50	4.22	0.66	0.00
59879	ADL	SEG	w	2	6.79	1.07	0.05	0.16	0.16	0.66	10.51	219.88	5.11	0.10	0.75	76.60	18.81	0.39	0.44	20.70	6.05	2.55	0.00
59880	RES	SEG	m	2	5.06	2.93	0.06	0.26	0.22	0.67	7.73	142.53	4.90	0.10	3.73	66.20	16.98	0.29	0.32	22.90	4.68	2.31	0.00
59881	ADL	SEG	m	2	6.40	2.00	0.01	0.26	0.25	0.56	18.51	183.49	9.93	0.57	3.69	64.40	14.50	0.31	0.49	18.50	5.37	1.60	0.00
59882	RES	SEG	w	2	4.24	3.62	0.01	0.47	0.18	0.54	15.02	139.03	9.66	1.53	5.84	58.30	18.07	0.19	0.64	19.30	6.25		0.00
59883	ADL	SEG	w	2	0.06	0.01	0.01	0.02	0.03	0.01	13.19	161.96	12.09	1.41	6.32	0.20	4.18	0.38	0.68	21.50	3.27	0.96	0.00
59885	ADL	SEG	w	2	5.35	2.32	0.03	0.43	0.28	0.60	22.51	156.92	4.80	0.83	3.95	63.40	20.05	0.38	0.39	19.40	5.37	1.89	0.00
59886	RES	SEG	m	2	5.99	2.01	0.01	0.23	0.28	0.93	18.16	175.43	4.62	0.10	2.17	65.20	16.67	0.44	0.25	19.90	3.23		0.00
59857	RES	HF	m	8	6.45	0.63	0.01	0.05	0.24	1.69	34.86	101.68	3.46	0.48	1.73	60.00	16.98	0.09	0.15	19.80	2.97	1.20	0.00
59858	RES	SEG	w	8	5.13	0.61	0.01	0.13	0.19	1.41	14.24	88.97	13.96	4.76	10.98	52.80	10.47	0.12	0.09	22.00	5.55	3.13	0.00
59859	RES	SEG	m	8	5.66	0.65	0.03	0.09	0.15	0.99	24.39	77.17	1.54	0.78	1.02	48.90	4.55		0.39	20.10	4.89	0.00	0.00
59860	RES	SEG	m	8	5.80	0.55	0.04	0.08	0.40	1.54	9.59	67.59	12.52	1.21	5.75	57.90	10.08	0.22	0.28	19.60	1.06	0.60	0.00
59861	RES	SEG	w	8	3.11	0.68	0.13	0.21	0.12	1.34	6.81	20.00	2.34	1.75	2.41	46.40	5.91	0.32	0.37	18.50	10.52	5.66	0.00
59862	ADL	SEG	m	8	7.37	0.50	0.01	0.07	0.11	1.37	19.97	59.26	3.23	0.43	1.09	51.70	5.98	0.15	0.00	21.70	3.28	6.12	0.00
59863	ADL	HF	w	8	6.15	0.86	0.02	0.15	0.68	1.20	20.32	20.00	5.01	2.03	2.56	47.40	3.82	0.25	0.22	21.50	4.26	4.00	0.00
59864	ADL	SEG	m	8	5.04	0.62	0.03	0.14	0.15	1.59	11.52	70.95	1.20	0.71	1.66	59.70	9.26	0.37	0.31	22.90	2.17	1.87	0.00
59866	ADL	SEG	w	8	7.29	0.56	0.01	0.04	0.16	1.26	49.18	20.00	5.30	1.83	3.04	42.30	7.94	0.40	0.61	20.00	4.04	2.08	0.00
59867	ADL	SEG	m	8	4.39	0.05	0.02	0.08	0.16	1.20	21.92	59.48	3.60	0.75	2.00	45.90	13.35	0.26	0.45	19.60	5.15	0.92	0.00
59868	RES	SEG	m	8	4.15	0.91	0.02	0.13	0.53	1.78	14.00	20.00	2.32	1.05	2.40	45.00	15.21	0.11	0.09	21.10	4.40	2.08	0.00
59869	ADL	SEG	m	8	5.73	0.31	0.03	0.07	0.11	1.50	17.77	44.51	7.80	3.52	10.56	54.40	9.28	0.09	0.30	20.70	6.60		0.00
59870	RES	SEG	m	8	4.64	0.81	0.01	0.06	0.29	1.70	86.46	20.00	1.58	0.88	1.45	49.30	5.43	0.23	0.34	22.80	6.71	2.36	0.00
59871	ADL	HF	m	8	6.77	0.60	0.01	0.06	0.24	1.35	44.47	77.90	3.22	0.10	1.67	48.60	11.92	0.19	0.11	21.10	2.24	2.73	0.00
59872	RES	SEG	m	8	4.84	0.60	0.01	0.17	0.21	1.02	4.20	62.72	3.89	0.85	1.82	54.30	11.20	0.13	0.08	20.90	6.56	0.96	0.00
59873	ADL	SEG	m	8	5.46	0.62	0.01	0.07	0.20	0.87	10.95	128.41	8.99	0.10	1.97	60.00	16.78	0.12	0.89	20.80	2.41	2.00	0.00
59874	RES	SEG	m	8	4.46	0.42	0.02	0.09	0.15	1.10	15.09	20.00	0.89	0.68	0.79	56.20	16.98	0.35	0.34	18.70	6.65	2.04	0.00
59875	ADL	HF	m	8	5.79	0.69	0.01	0.26	0.26	1.27	3.14	20.00	1.72	1.42	1.88	54.10	14.08	0.37	0.30	20.40	4.97	2.54	0.00
59876	RES	HF	m	8	5.24	0.59	0.01	0.11	0.18	1.17	12.84	32.00	2.01	0.97	1.76	58.00	10.70	0.17	0.31	21.40	4.65	2.55	0.00
59877	ADL	SEG	w	8	7.97	0.43	0.03	0.15	0.09	0.71	9.32	20.00	1.27	0.71	1.30	60.70	9.89	0.18	0.61	20.80	5.84	5.00	0.08
59878	RES	SEG	m	8	5.60	0.34	0.01	0.20	0.24	1.32	11.13	20.00	2.56	0.10	0.56	53.40	19.65	0.12		19.60	5.94	0.38	0.00
59879	ADL	SEG	w	8	7.68	0.47	0.02	0.18	0.20	1.29	10.06	79.59	3.92	0.10	1.54	69.40	10.76	0.24	0.36	21.30	2.46	5.21	0.00
59880	RES	SEG	m	8	4.67	0.65	0.01	0.14	0.12	1.22	8.66	61.66	1.35	0.10	1.58	52.90	11.67	0.26	0.29	21.80	3.53	2.72	0.00
59881	ADL	SEG	m	8	4.90	0.67	0.01	0.15	0.12	0.94	2.51	20.00	3.13	1.50	1.36	54.50	5.52	0.13	0.31	19.60	6.28	10.00	0.00
59882	RES	SEG	w	8	5.63	0.59	0.02	0.07	0.23	0.85	19.54	49.67	3.74	3.80	4.40	53.20	10.76	0.38	0.13	18.40	7.46	5.60	0.00
59883	ADL	SEG	w	8	5.54	0.47	0.02	0.05	0.19	1.17	26.83	20.00	3.84	1.27	1.83	39.90	2.52	0.17	0.44	19.60	3.46	1.43	0.00
59885	ADL	SEG	w	8	7.60	0.41	0.02	0.09	0.19	1.11	10.77	59.56	1.21	0.72	1.72	54.80	21.36	0.28	0.36	18.70	5.18	3.51	0.00
59886	RES	SEG	m	8	4.49	0.57	0.01	0.05	0.11	1.45	7.66	64.08	1.58	0.79	1.13	57.00	9.52	0.40	0.14	20.70	4.30		0.00
59857	RES	HF	m	15	8.84	0.72	0.03	0.12	0.34	2.65	14.78	268.15	5.33	0.72	1.52	72.80	16.28	0.38	0.97	27.30	2.03	2.41	0.00
59858	RES	SEG	w	15	6.71	1.29	0.01	0.17	0.23	2.31	23.18	103.80	11.85	3.01	8.39	55.80	9.32	0.18	0.48	25.70	2.58	3.15	0.00
59859	RES	SEG	m	15	6.04	0.68	0.01	0.09	0.23	2.04	8.31	139.79	0.94	1.42	1.39	52.90	4.80	0.24	0.58	23.20	1.91	1.72	0.00

Calf	Group	Breed	Sex	Day of life	Glucose (mmol/L)	Lactate (mmol/L)	BHB (mmol/L)	NEFA (mmol/L)	Triglycerides (mmol/L)	Cholesterol (mmol/L)	Insulin (uU/mL)	IGF-I (µg/L)	IGFBP3 (µg/L)	IGFBP2 (µg/L)	IGFBP4 (µg/L)	Total protein (g/L)	IgG1 (g/L)	IgG2 (g/L)	IgM (g/L)	Albumin (g/L)	Urea (mmol/L)	Fibrinoge (g/L)	Haptoglobin (g/L)
59860	RES	SEG	m	15	6.14	0.54	0.04	0.05	0.22	1.87	9.96	81.80	18.06	4.63	12.02	57.40	7.98	0.28	0.49	22.00	1.41	0.50	0.00
59861	RES	SEG	w	15	3.94	0.64	0.03	0.12	0.20	1.38	3.94	52.29	3.73	1.83	2.41	53.50	5.91	0.32	0.39	22.20	2.56	7.82	0.00
59862	ADL	SEG	m	15	3.78	0.62	0.01	0.11	0.68	1.82	5.27	93.60	3.83	0.65	1.14	54.00	5.98	0.21	0.47	25.20	1.16	4.44	0.00
59863	ADL	HF	w	15	9.39	1.61	0.02	0.09	0.83	2.64	148.74	138.62	7.33	1.07	1.75	58.90	5.32	0.22	0.43	26.20	0.94	4.35	0.00
59864	ADL	SEG	m	15	7.10	1.47	0.01	0.02	0.87	2.73	52.94	98.49	2.04	0.61	1.59	57.90	7.49	0.35	0.83	23.80	0.78	5.76	0.00
59866	ADL	SEG	w	15	4.40	0.56	0.01	0.09	0.27	1.74	15.01	20.00	3.55	2.04	1.39	43.70	7.16	0.30	1.17	22.10	1.34	4.63	0.00
59867	ADL	SEG	m	15	4.61	0.05	0.04	0.08	0.12	0.90	22.54	55.69	3.44	1.19	1.69	49.50	12.98	0.24	0.66	22.70	3.51	3.51	0.00
59868	RES	SEG	m	15	4.08	1.11	0.09	0.19	0.11	1.25	15.40	20.00	6.86	5.67	8.17	53.20	14.08	0.19	0.40	24.80	5.71	12.76	0.00
59869	ADL	SEG	m	15	3.81	0.01	0.02	0.14	0.11		15.18	20.00	3.49	1.44	4.91		6.93	0.15	0.26	20.90	5.74	5.76	0.66
59870	RES	SEG	m	15	6.25	1.23	0.01	0.08	0.18	1.52	13.35	20.00	2.32	0.66	1.94	52.70	4.51	0.24	0.88	25.40	1.72	7.08	0.00
59871	ADL	HF	m	15	5.13	0.45	0.01	0.08	0.36	0.88	13.45	68.53	2.71	0.10	1.05	53.70	10.88	0.42	1.60	22.80	1.14	8.60	0.00
59872	RES	SEG	m	15	4.29	0.60	0.06	0.17	0.21	1.67	4.75	56.24	0.00	1.79	1.42	54.30	7.60	0.25	0.48	22.10	5.30	0.82	0.00
59873	ADL	SEG	m	15	5.96	0.77	0.01	0.07	0.63	1.51	10.12	132.01	8.23	0.72	1.13	56.50	15.21	0.21	0.38	21.60	1.07	3.85	0.00
59874	RES	SEG	m	15	4.85	1.02	0.01	0.15	0.16	1.49	2.83	32.00	1.26	1.20	1.24	58.30	12.93	0.27	1.27	21.80	2.98	4.00	0.00
59875	ADL	HF	m	15	4.56	1.22	0.14	0.01	0.28	1.10	7.91	20.00	1.11	0.95	1.72	55.40	13.35	0.32	0.48	23.10	2.46	4.09	0.00
59876	RES	HF	m	15	6.92	0.75	0.02	0.15	0.31	1.54	21.29	49.18	2.22	0.97	1.43	57.50	9.63	0.25	0.54	23.70	2.73	7.35	0.00
59877	ADL	SEG	w	15	8.63	1.43	0.01	0.18	0.94	1.74		20.00	2.71	0.10	1.76	63.10	10.47	0.31	0.82	23.30	0.72	2.86	0.00
59878	RES	SEG	m	15	4.24	0.51	0.04	0.21	0.11	0.88	7.13	20.00	0.99	1.95	0.10	44.20	9.88	0.19	0.87	19.60	4.97	3.39	0.00
59879	ADL	SEG	w	15	8.69	0.70	0.01	0.08	0.30	0.78	15.02	66.01	4.08	0.10	0.65	57.20	9.88	0.34	1.37	20.90	1.56	2.94	0.00
59880	RES	SEG	m	15	3.37	0.50	0.01	0.13	0.09	1.13	2.65	20.00	1.64	1.74	2.94	48.20	9.60	0.16	0.49	21.60	3.22	5.76	0.00
59881	ADL	SEG	m	15	6.66	1.00	0.01	0.10	0.53	1.45	10.70	88.29	3.87	0.69	1.36	54.00	5.23	0.23	0.31	21.90	1.18	6.40	0.00
59882	RES	SEG	w	15	5.93	0.71	0.01	0.01	0.20	1.39	13.97	88.45	11.24	3.82	7.17	52.80	11.06	0.30	1.38	20.70	1.70	9.63	0.00
59883	ADL	SEG	w	15	6.05	0.61	0.03	0.08	0.11	1.62	1.76	51.08	6.94	3.55	3.16	48.10	2.79	0.29	0.65	23.90	2.78	3.09	0.00
59885	ADL	SEG	w	15	8.45	0.66	0.02	0.13	0.40	1.69	5.58	88.01	2.09	0.63	1.69	59.70	17.98	0.29	0.34	22.20	0.73	4.76	0.00
59886	RES	SEG	m	15	3.96	0.42	0.02	0.09	0.27	1.75	11.26	20.00	0.96	1.39	1.48	52.30	6.97	0.29	0.97	21.00	3.30	8.51	0.00
59857	RES	HF	m	22	5.85	0.45	0.01	0.13	0.86	2.54	11.17	286.88	7.37	0.99	2.36	62.10	15.93	0.37	1.39	25.50	1.73	1.45	0.00
59858	RES	SEG	w	22	7.36	0.52	0.01	0.13	0.20	3.57	20.63	100.64	13.94	3.76	6.79	60.20	9.03	0.20	0.31	28.50	2.80	3.00	0.00
59859	RES	SEG	m	22	6.73	0.57	0.04	0.10	0.10	2.16	14.63	107.69	1.14	1.32	1.45	48.60	4.06	0.22	0.41	22.40	2.47	4.63	0.00
59860	RES	SEG	m	22	5.67	0.80	0.05	0.12	0.30	2.10	8.83	81.27	17.01	4.44	9.20	52.50	6.99	0.24	0.24	22.10	1.33	3.92	0.00
59861	RES	SEG	w	22	4.80	0.66	0.03	0.10	0.28	2.44	10.95	90.90	7.16	1.10	2.62	50.80	5.91	0.29	0.33	22.60	1.25	4.72	0.00
59862	ADL	SEG	m	22	7.29	0.70	0.01	0.14	0.65	2.42	144.06	250.75	6.28	0.10	1.60	47.80	5.98	0.13	0.16	24.20	0.95	3.83	0.00
59863	ADL	HF	w	22	4.31	0.82	0.01	0.15	0.56	3.29	18.60	244.71	9.88	1.03	2.17	48.60	4.30	0.20	0.39	23.40	1.01	0.00	0.00
59864	ADL	SEG	m	22	6.64	0.61	0.02	0.08	0.54	2.01	49.54	102.85	1.28	0.63	1.47	52.10	7.49	0.36	0.60	23.60	1.01	9.80	0.00
59866	ADL	SEG	w	22	4.46	0.47		0.02	0.10	0.77	27.14	47.38	5.74	1.09	2.34	46.30	6.39	0.02		22.80	1.84	3.85	0.00
59867	ADL	SEG	m	22	5.33	0.05	0.01	0.10	0.91	1.84	58.18	73.46	6.66	1.41	3.79	49.10	10.88	0.22	0.47	22.90	1.69	0.96	0.00
59868	RES	SEG	m	22	4.47	0.49	0.04	0.32	0.18	1.89	11.68	20.00	3.29	1.81	3.18	50.70	13.35	0.18	0.40	25.00	3.46	9.76	0.00
59869	ADL	SEG	m	22	4.05	0.41	0.08	0.18	0.23	1.81	14.86	50.93	1.74	0.82	1.93	49.70	6.29	0.17	0.19	22.30	2.53	5.76	0.00
59870	RES	SEG	m	22	7.79	0.62	0.01	0.10	0.48	1.62	41.52	54.06	4.50	1.49	3.48	46.60	3.64	0.19	0.57	24.10	1.12	8.33	0.00
59871	ADL	HF	m	22	5.17	0.61	0.01	0.09	0.32	1.74	63.44	175.92	6.97	0.10	2.18	48.40	9.64	0.41	0.65	23.10	0.86	3.78	0.00
59872	RES	SEG	m	22	4.77	0.42	0.02	0.08	0.08	1.59	23.18	33.00	1.97	1.00	1.50	52.80	7.27	0.34	0.26	22.30	2.76	9.00	0.00
59873	ADL	SEG	m	22	6.07	0.86	0.01	0.07	0.37	1.45	40.21	199.49	8.17	0.10	1.34	53.40	14.44	0.25	0.21	22.30	0.91	3.12	0.00
59874	RES	SEG	m	22	4.15	0.47	0.08	0.23	0.37	1.91	3.09	20.00	0.77	0.83	1.31	53.50	11.36	0.26	0.74	21.20	1.91	8.23	0.00
59875	ADL	HF	m	22	5.08	0.85	0.03	0.09	0.10	1.26	7.04	46.90	1.56	0.91	1.27	53.40	11.92	0.31	0.44	23.60	2.47	3.28	0.00
59876	RES	HF	m	22	5.13	0.76	0.01	0.17	0.52	1.80	7.38	62.15	2.33	0.82	1.27	54.70	9.63	0.26	0.23	23.80	1.63	6.52	0.00
59877	ADL	SEG	w	22	8.76	2.63	0.03	0.13	0.78	2.49	10.78	158.79	5.03	0.10	2.34	61.00	9.03	0.29	0.43	24.20	0.97	3.56	0.00
59878	RES	SEG	m	22	4.94	1.13	0.03	0.09	0.57	2.25	14.74	52.68	3.76	1.07	0.60	49.40	9.88	0.22	0.34	21.40	1.91	3.92	0.00
59879	ADL	SEG	w	22	7.53	1.10	0.01	0.11	0.50	2.03	10.19	80.36	5.21	0.10	1.99	59.80	9.88	0.32	1.15	23.50	0.86	4.00	0.00
59880	RES	SEG	m	22	4.00	1.21	0.02	0.13	0.35	2.29	12.26	20.00	2.31	1.12	2.43	50.00	8.76	0.14	0.55	23.00	2.46	4.00	0.00
59881	ADL	SEG	m	22	5.80	1.17	0.01	0.10	0.67	1.94	44.83	102.15	4.36	0.61	1.24	51.30	5.23	0.21	0.35	22.20	1.22	2.32	0.00
59882	RES	SEG	w	22	5.80	0.71	0.01	0.16	0.26	2.87	7.26	111.79	7.65	2.40	3.76	54.10	10.47	0.33	0.74	22.30	1.16	7.50	0.00
59883	ADL	SEG	w	22	3.96	0.05	0.01	0.09	0.11	1.80	1.80	20.00	5.14	2.66	1.06	44.20	3.06	0.25	0.32	23.50	1.96	1.46	0.00
59885	ADL	SEG	w	22	7.48	1.47	0.02	0.10	0.23	1.84	9.34	81.94	1.30	0.66	1.57	53.40	17.16	0.25	0.16	22.40	1.29	7.02	0.00
59886	RES	SEG	m	22	3.96	0.33	0.02	0.08	0.20	2.91	10.48	41.58	1.14	1.30	1.55	53.50	6.64	0.28	0.45	22.90	2.34	8.00	0.00
59857	RES	HF	m	29	4.87	0.48	0.26	0.38	0.16	2.48	2.61	169.36	4.75	1.28	1.77	59.40	15.24	0.35	0.74	25.90	2.40	4.00	0.00
59858	RES	SEG	w	29	5.77	1.07	0.01	0.15	0.30	4.07	7.93	163.14	11.04	2.31	4.34	56.20	9.32	0.21	0.27	26.70	2.44	3.06	0.00
59859	RES	SEG	m	29	4.78	0.40	0.08	0.28	0.11	2.57	2.33	109.51	1.09	1.28	1.27	52.50	3.10	0.21	0.34	24.50	2.27	2.83	0.00
59860	RES	SEG	m	29	4.50	0.47	0.05	0.26	0.28	2.47	3.47	94.62	8.10	0.99	2.14	51.30	6.39	0.40	0.22	23.20	1.46	3.84	0.00
59861	RES	SEG	w	29	3.97	0.42	0.04	0.23	0.15	2.57	2.07	87.31	5.84	1.99	1.99	47.80	5.91	0.29	0.36	23.30	2.04	2.94	0.00
59862	ADL	SEG	m	29	6.59	0.52	0.01	0.12	0.41	2.31	50.19	336.60	7.42	0.50	2.38	47.40	4.55	0.12	0.18	24.40	1.17	3.67	0.00
59863	ADL	HF	w	29	7.11	0.47	0.01	0.14	0.45	1.96	20.64	153.46	8.52	1.41	1.59	48.50	4.06	0.19	0.67	24.80	1.47	3.46	0.00
59864	ADL	SEG	m	29	4.86	0.37	0.04	0.05	0.25	2.80	78.98	110.77	3.08	0.10	2.09	51.50	7.21	0.29	0.58	23.60	1.00	4.89	0.00
59866	ADL	SEG	w	29	5.95	0.64	0.01	0.11	1.22	3.47	129.18	115.81	6.79	0.48	1.58	46.90	6.90	0.28	0.44	23.50	0.77	2.00	0.00
59867	ADL	SEG	m	29	4.61	1.27	0.03	0.12	1.03	2.75	59.87	80.51	4.87	1.19	2.43	46.50	9.88	0.22	0.35	22.50	1.59	5.78	0.00
59868	RES	SEG	m	29	0.02	1.25	0.05	0.08	0.22	2.23	15.11	20.00	6.53	4.20	8.13	51.90	13.35	0.23	0.27	25.00	2.51	11.06	0.00
59869	ADL	SEG	m	29	6.76	0.83	0.02	0.02	0.54	2.53	129.33	101.92	2.14	0.43	3.46	46.00	4.76	0.16	0.20	21.90	1.27	1.88	0.00
59870	RES	SEG	m	29	6.47	0.51	0.02	0.10	0.24	2.67	63.09	85.70	2.76	0.69	2.01	48.10	3.64	0.19	0.45	25.70	1.58	3.57	0.00
59871	ADL	HF	m	29	7.70	0.45	0.01	0.14	0.55	1.89	56.31	323.11	19.27	0.80	5.52	48.80	8.60	0.41	0.64	23.80	1.07	2.55	0.00
59872	RES	SEG	m	29	5.32	0.43	0.04	0.12	0.30	2.59	10.74	98.50	2.65	0.57	1.20	54.30	5.50	0.23	0.30	23.60	2.09	8.51	0.00
59873	ADL	SEG	m	29	4.65	0.47	0.01	0.16	0.31	1.82	7.49	195.83	10.02	0.80	1.49	51.60	14.44	0.24	0.20	22.90	1.76	2.31	0.00
59874	RES	SEG	m	29	4.64	0.39	0.05	0.18	0.28	2.53	22.13	20.00	1.04	1.26	1.19	54.50	10.18	0.24	0.65	23.10	3.79	4.60	0.03
59875	ADL	HF	m	29	7.88	0.67	0.01	0.16	0.83	2.52	99.35	111.30	3.23	0.10	1.76	49.90	11.56	0.30	0.37	23.10	1.24		0.00
59876	RES	HF	m	29	5.93	0.05	0.02	0.15	0.70	2.78	13.38	103.71	4.88	0.99	2.38	54.80	9.28	0.37	0.27	24.50	1.53	3.84	0.00
59877	ADL	SEG	w	29	8.70	1.16	0.01	0.09	0.69	2.45	66.18	194.96	8.12	0.10	3.13	55.30	7.68	0.25	0.25	23.40	1.20	1.43	0.00
59878	RES	SEG	m	29	4.58	0.41	0.06	0.14	0.23	2.90	14.34	43.38	3.37	1.33	0.73	46.40	8.60	0.18	0.30	21.70	2.30	1.96	0.00
59879	ADL	SEG	w	29	6.89	0.58	0.01	0.12	0.43	3.23	11.58	147.83	6.53	0.10	3.56	59.70	9.03	0.29	0.77	24.00	1.32	4.09	0.00
59880	RES	SEG	m	29	3.91	0.57	0.08	0.09	0.33	3.52	6.16	61.88	2.27	0.87	1.89	51.90	8.76	0.14	0.73	24.60	2.47	6.78	0.00
59881	ADL	SEG	m	29	5.11	0.45	0.01	0.10	0.31	2.11	7.27	100.19	5.92	1.16	1.76	50.10	3.33	0.22	0.36	22.50	2.59	10.71	0.00
59882	RES	SEG	w	29	5.25	0.80	0.07	0.12	0.34	2.32	15.91	89.22	12.59	3.87	5.78	53.70	9.88	0.35	0.62	22.30	2.66	8.57	0.00
59883	ADL	SEG	w	29	3.61	0.74	0.01	0.09	0.38	2.95	21.44	85.31	20.92	3.71	7.87	42.10	3.06	0.21	0.24	22.90	0.61	0.71	0.00
59885	ADL	SEG	w	29	7.82	1.30	0.02	0.07	0.50	2.84	35.39	174.22	3.15	0.10	2.18	54.50	15.21	0.23	0.23	24.00	1.38	4.16	0.00
59886	RES	SEG	m	29	3.81	0.36	0.08	0.07	0.20	3.05	20.47	41.12	1.08	1.26	1.37	52.60	6.97	0.27	0.44	23.20	2.24	6.89	0.00
59857	RES	HF	m	36	4.93	0.34	0.05	0.15	0.18	3.18	4.04	307.21	7.14	1.19	2.28	55.00	13.90	0.34	0.44	25.90	1.56	2.50	0.00
59858	RES	SEG	w	36	6.07	1.76	0.08	0.28	0.27	5.51	13.42	161.01	11.66	3.16	3.99	70.30	8.76	0.22	0.62	33.90	2.37	1.36	0.00
59859	RES	SEG	m	36	4.40	0.37	0.05	0.15	0.14	3.07	2.31	126.46	1.88	1.48	1.52	50.60	2.87	0.22	0.32	24.80	2.51		0.00
59860	RES	SEG	m	36	5.17	0.52	0.08	0.12	0.28	2.94	9.30	88.16	13.59	2.98	6.24	53.00	6.99	0.23	0.24	25.40	1.54	2.78	0.00
59861	RES	SEG	w	36	4.11	0.32	0.06	0.12	0.14	2.91	2.43	70.84	5.40	1.77	2.87	47.70	5.42	0.31	0.33	23.20	1.89	2.10	0.00
59862	ADL	SEG	m	36	6.88	0.66	0.04	0.20	0.38	1.20	73.83	89.95	4.97	1.24	1.04	43.50	4.80	0.00	0.00	23.90	1.36	1.96	0.00

Calf	Group	Breed	Sex	Day of life	Glucose (mmol/L)	Lactate (mmol/L)	BHB (mmol/L)	NEFA (mmol/L)	Triglycerides (mmol/L)	Cholesterol (mmol/L)	Insulin (uU/mL)	IGF-I (µg/L)	IGFBP3 (µg/L)	IGFBP2 (µg/L)	IGFBP4 (µg/L)	Total protein (g/L)	IgG1 (g/L)	IgG2 (g/L)	IgM (g/L)	Albumin (g/L)	Urea (mmol/L)	Fibrinoge (g/L)	Haptoglobin (g/L)
59863	ADL	HF	w	36	5.28	0.05	0.01	0.09	0.46	3.61	37.21	307.23	10.65	1.11	2.30	49.20	3.58	0.22	0.61	25.70	1.06	1.96	0.00
59864	ADL	SEG	m	36	5.02	0.36	0.04	0.14	0.53	3.35	23.78	158.33	1.80	0.84	2.25	51.20	5.32	0.20	0.47	24.10	1.11	3.12	0.00
59866	ADL	SEG	w	36	6.41	0.53	0.04	0.10	0.39	3.54	60.53	188.54	10.49	1.05	3.56	45.20	6.39	0.30	0.58	23.30	0.76	1.92	0.00
59867	ADL	SEG	m	36	3.99	0.81	0.06	0.22	0.26	3.09	18.07	133.36	8.93	1.68	3.11	47.10	8.28	0.22	0.29	24.20	1.70	3.51	0.00
59868	RES	SEG	m	36	3.66	1.77	0.07	0.05	0.32	2.81	13.75	51.78	2.24	1.39	3.07	53.70	13.11	0.23	0.31	26.40	2.46	8.00	0.00
59869	ADL	SEG	m	36	4.03	1.56	0.01	0.03	0.33		43.21	183.32	2.82	0.10	2.92	46.90	4.76	0.19	0.08		0.13	1.92	0.00
59870	RES	SEG	m	36	4.63	0.35	0.03	0.17	0.26	3.05	8.39	86.50	2.53	1.05	1.67	49.80	4.07	0.19	0.58	26.10	2.04	5.88	0.00
59871	ADL	HF	m	36	5.71	0.41	0.01	0.18	0.31	1.65	19.80	148.13	15.56	2.99	4.87	48.60	6.20	0.26	0.89	24.40	1.58	1.82	0.00
59872	RES	SEG	m	36	4.23	0.45	0.03	0.12	0.26	2.93	8.60	111.33	6.70	0.93	2.05	50.70	6.67	0.21	0.25	23.50	2.01	4.09	0.00
59873	ADL	SEG	m	36	6.92	0.54	0.01	0.11	0.41	2.37	51.46	191.46	10.02	0.89	1.27	53.10	14.08	0.25	0.21	24.20	1.80	2.00	0.00
59874	RES	SEG	m	36	4.39	0.34	0.04	0.04	0.13	2.94	25.63	45.16	0.80	0.77	0.79	52.10	10.18	0.22	0.62	22.90	2.42	3.00	0.00
59875	ADL	HF	m	36	6.88	0.64	0.01	0.20	0.64	2.77	28.04	155.41	4.60	0.10	1.79	53.00	11.56	0.31	0.35	24.10	0.95	1.62	0.00
59876	RES	HF	m	36	5.32	0.05	0.02	0.13	0.41	2.92	12.11	106.84	3.23	0.88	1.31	54.70	9.28	0.38	0.25	24.70	2.11	9.43	0.00
59877	ADL	SEG	w	36	7.49	0.64	0.01	0.07	0.40	2.76	120.94	204.22	4.54	0.10	1.10	53.30	7.94	0.31	0.24	23.90	1.35	4.29	0.00
59878	RES	SEG	m	36	3.78	0.35	0.16	0.18	0.26	2.94	6.69	20.00	2.29	1.73	0.61	48.20	8.91	0.20	0.16	22.80	2.41	6.66	0.00
59879	ADL	SEG	w	36	8.50	1.09	0.01	0.07	0.51	3.84	24.99	194.63	6.62	0.10	2.20	57.10	6.64	0.22	0.74	23.50	1.56	5.40	0.00
59880	RES	SEG	m	36	3.84	0.40	0.05	0.08	0.34	3.23	14.10	71.19	2.99	0.93	1.82	51.10	8.43	0.14	1.25	24.60	2.39		0.00
59881	ADL	SEG	m	36	5.92	0.43	0.01	0.19	0.46	2.47	34.26	96.88	5.04	1.47	1.23	52.90	3.33	0.18	0.18	24.70	2.02	5.66	0.00
59882	RES	SEG	w	36	4.46	0.34	0.03	0.10	0.16	3.20	2.70	93.44	23.52	9.35	14.26	52.40	10.18	0.34	0.50	22.50	1.67	3.92	0.00
59883	ADL	SEG	w	36	4.93	0.55	0.01	0.17	0.53	2.67	41.26	105.63	7.03	1.38	1.70	42.70	3.06	0.21	0.27	23.40	1.15	0.41	0.00
59885	ADL	SEG	w	36	7.90	0.44	0.03	0.20	0.21	2.46	13.71	143.56	1.85	0.83	2.33	54.40	9.88	0.27	0.31	24.40	1.40	3.78	0.00
59886	RES	SEG	m	36	2.87	0.26	0.08	0.17	0.25	3.49	2.56	48.19	1.94	1.45	1.61	57.00	6.69	0.25	0.44	25.20	3.47	9.61	0.00
59857	RES	HF	m	43	5.42	0.56	0.05	0.15	0.33	3.18	24.19	251.07	7.70	1.09	2.20	55.30	14.22	0.35	0.41	26.90	1.74	2.40	0.00
59858	RES	SEG	w	43	6.95	0.55	0.08	0.21	0.29	4.79	42.46	136.97	15.44	5.63	7.09	60.00	6.96	0.21	0.43	30.70	1.97	1.23	0.00
59859	RES	SEG	m	43	5.60	0.35	0.11	0.12	0.32	3.40	24.23	109.47	1.30	1.02	1.81	48.20	3.10	0.21	0.41	24.60	1.74	0.89	0.00
59860	RES	SEG	m	43	3.90	0.43	0.08	0.27	0.11	2.60	8.85	83.70	15.67	5.88	11.82	48.60	4.67	0.24	0.35	24.20	2.38	2.08	0.00
59861	RES	SEG	w	43	2.81	0.54	0.20	0.27	0.09	1.79	2.06	20.00	2.64	2.17	2.49	48.40	5.42	0.34	0.27	24.20	3.39	4.79	0.00
59862	ADL	SEG	m	43	5.00	0.47	0.12	0.26	0.21	2.39	11.54	104.39	6.30	4.12	3.82	48.30	4.55	0.17	0.39	25.00	2.12	3.77	0.33
59863	ADL	HF	w	43	5.25	0.81	0.05	0.15	0.28	3.36	22.04	201.01	9.31	1.88	2.18	52.70	3.58	0.29	0.68	27.10	1.87		0.00
59864	ADL	SEG	m	43	0.56	0.51	0.13	0.22	0.34		14.16	153.21	1.29	1.54	1.58	50.50	4.55	0.27	0.77	25.60	1.27	2.35	0.00
59866	ADL	SEG	w	43	4.53	0.48	0.09	0.27	0.23	3.64	15.25	136.59	9.33	2.44	3.01	47.40	7.16	0.32	0.61	24.60	2.24	2.58	0.00
59867	ADL	SEG	m	43	4.27	0.43	0.17	0.25	0.69	3.03	20.34	143.59	7.30	1.88	3.02	45.00	7.66	0.26	0.49	24.00	2.03	2.83	0.00
59868	RES	SEG	m	43	4.32	0.40	0.01	0.12	0.32	2.40	10.96	73.59	2.28	0.61	1.81	47.00	10.88	0.24	0.21	24.50	2.26	4.42	0.00
59869	ADL	SEG	m	43	4.75	0.34	0.07	0.28	0.21	2.97	12.56	99.22	2.86	1.17	3.00	48.20	5.06	0.16	0.11	24.40	2.42	1.11	0.00
59870	RES	SEG	m	43	5.82	0.42	0.03	0.17	0.72	3.44	83.71	134.31	5.95	0.85	2.87	47.60	3.23	0.21	0.53	25.90	1.26	3.33	0.00
59871	ADL	HF	m	43	4.67	0.47	0.06	0.33	0.27	2.73	5.92	177.89	10.26	1.09	2.46	49.60	6.20	0.46	0.84	25.50	1.26	2.86	0.00
59872	RES	SEG	m	43	5.08	0.49	0.09	0.14	0.32	3.07	16.21	92.94	6.01	1.01	1.29	51.60	6.67	0.22	0.30	24.60	3.41	5.21	0.00
59873	ADL	SEG	m	43	4.89	0.32	0.04	0.15	0.26	2.78	10.61	140.96	9.87	1.68	1.70	54.10	11.56	0.24	0.24	25.60	2.44	2.00	0.00
59874	RES	SEG	m	43	3.86	0.31	0.10	0.09	0.24	2.87	6.90	55.46	1.64	1.27	1.73	51.70	9.89	0.29	0.68	23.30	3.00	6.86	0.00
59875	ADL	HF	m	43	4.24	0.44	0.04	0.25	0.15	3.03	2.17	122.45	2.75	1.13	0.89	49.80	9.23	0.33	0.38	24.30	1.29	1.06	0.00
59876	RES	HF	m	43	5.23	0.05	0.15	0.27	0.51	3.51	10.91	84.19	3.90	1.36	2.02	55.80	9.63	0.28	0.28	25.90	1.94	3.07	0.00
59877	ADL	SEG	w	43	5.47	0.67	0.10	0.24	0.19	2.70	18.34	117.72	3.90	1.45	1.72	55.30	7.16	0.24	0.34	26.00	3.31	6.89	0.00
59878	RES	SEG	m	43	4.05	0.24	0.10	0.07	0.23	3.31	5.05	45.67	2.57	1.24	0.64	49.50	9.23	0.17	0.20	23.50	2.25	3.51	0.00
59879	ADL	SEG	w	43	6.40	0.45	0.06	0.27	0.23	3.54	2.68	152.48	5.67	0.74	1.55	55.60	7.15	0.26	0.91	24.80	1.86	4.79	0.00
59880	RES	SEG	m	43	4.78	0.43	0.21	0.10	0.36	2.49	26.87	94.69	3.16	1.04	2.38	49.30	8.76	0.15	1.26	23.80	2.66	5.00	0.00
59881	ADL	SEG	m	43	4.34	0.45	0.10	0.18	0.19	2.39	3.85	80.23	4.46	2.19	1.63	50.90	3.60	0.19	0.34	25.00	3.63	3.85	0.00
59882	RES	SEG	w	43	4.19	0.43	0.05	0.10	0.18	3.58	11.71	97.93	6.77	1.84	3.31	54.40	10.47	0.37	0.59	23.40	1.99	7.55	0.00
59883	ADL	SEG	w	43	4.42	0.50	0.05	0.20	0.23	2.44	12.57	63.89	2.90	2.75	1.48	50.30	3.33	0.29	0.55	25.60	3.37	2.55	0.00
59885	ADL	SEG	w	43	6.64	0.44	0.09	0.20	0.18	2.44	13.97	66.27	1.31	1.51	1.68	52.60	10.88	0.32	0.30	24.10	3.58	7.14	0.00
59886	RES	SEG	m	43	3.24	0.49	0.13	0.10	0.19	3.58	8.11	56.34	1.33	1.01	1.89	56.60	7.60	0.45	0.55	25.00	2.42	9.61	0.00
59857	RES	HF	m	50	5.29	0.44	0.01	0.15	0.38	3.34	17.24	304.64	7.75	1.44	2.74	53.40	15.24	0.26	0.92	27.10	1.88	1.37	0.00
59858	RES	SEG	w	50	4.92	0.56	0.11	0.07	0.22	4.26	7.65	171.62	7.26	1.45	2.50	54.40	7.16	0.26	0.19	27.90	2.04	2.50	0.00
59859	RES	SEG	m	50	5.21	0.30	0.15	0.06	0.15	3.44	5.97	206.48	2.75	1.49	2.29	47.60	6.11	0.48	0.43	25.50	2.16	1.15	0.00
59860	RES	SEG	m	50	5.27	0.44	0.20	0.07	0.33	2.57	4.07	134.79	14.81	5.03	6.11	51.10	3.59	0.23	0.35	25.60	1.16	1.86	0.00
59861	RES	SEG	w	50	4.27	0.35	0.19	0.04	0.30	2.36	9.47	75.69	8.21	3.23	4.23	50.40	5.91	0.37	0.43	24.80	1.73	4.60	0.00
59862	ADL	SEG	m	50	5.46	0.43	0.14	0.09	0.31	3.25	17.26	148.21	4.37	1.06	1.51	52.80	4.55	0.31	0.66	28.00	1.88	2.73	0.00
59863	ADL	HF	w	50	6.05	0.64	0.10	0.12	0.25	3.29	156.21	143.32	7.21	1.21	1.21	47.50	2.88	0.29	0.68	25.20	1.61	4.00	0.00
59864	ADL	SEG	m	50	3.91	0.36	0.16	0.17	0.35	4.99	18.69	155.33	1.52	1.11	1.46	51.10	3.57	0.30	0.61	26.70	1.74	0.57	0.00
59866	ADL	SEG	w	50	4.75	0.37	0.18	0.12	0.18	4.05	25.41	118.15	8.01	2.00	2.95	49.20	7.66	0.34	0.51	25.20	1.54	3.86	0.00
59867	ADL	SEG	m	50	4.02	0.44	0.20	0.20	0.21	3.23	12.86	132.25	3.04	0.99	1.41	47.30	7.66	0.26	0.57	25.10	1.82	3.75	0.00
59868	RES	SEG	m	50	3.95	0.44	0.04	0.02	0.18	2.36	21.91	86.99	1.18	0.58	1.38	45.50	11.91	0.26	0.21	23.40	2.14	3.33	0.00
59869	ADL	SEG	m	50	4.86	0.38	0.10	0.23	0.25	3.99	21.62	98.42	2.53	0.87	2.54		4.76	0.16	0.13	25.40	2.75	1.51	0.00
59870	RES	SEG	m	50	4.54	0.41	0.13	0.17	0.25	2.95	21.61	120.49	6.43	1.22	2.86	47.10	3.23	0.23	0.87	26.00	1.57	6.00	0.00
59871	ADL	HF	m	50	5.30	0.40	0.11	0.18	0.27	3.23	39.29	165.87	4.43	0.89	1.51	49.10		0.25	0.88	24.90	1.46	2.68	0.00
59872	RES	SEG	m	50	4.22	0.37	0.16	0.14	0.14	2.92	7.86	99.17	6.07	1.36	1.45	52.50	3.57	0.23	0.56	26.10	3.45	4.42	0.00
59873	ADL	SEG	m	50	5.41	0.40	0.08	0.17	0.16	2.33	57.68	75.27	8.86	2.33	1.94	48.60	7.67	0.23	0.17	23.10	2.73	7.84	0.00
59874	RES	SEG	m	50	3.27	0.32	0.12	0.08	0.13	3.06	3.07	20.00	1.13	2.50	1.48	49.80	9.60	0.32	0.73	23.10	4.28	2.45	0.00
59875	ADL	HF	m	50	4.37	0.43	0.08	0.31	0.17	3.61	2.07	113.66	2.76	1.40	1.53	51.00	10.88	0.47	0.40	25.90	1.40	1.00	0.00
59876	RES	HF	m	50	4.74	0.05	0.14	0.19	0.26	3.64	7.64	96.51	3.97	1.09	1.51	56.10	9.63	0.39	0.28	26.10	2.07	1.75	0.00
59877	ADL	SEG	w	50	5.29	0.39	0.13	0.14	0.14	2.53	31.19	74.88	3.56	1.55	2.55	53.60	6.90	0.33	0.49	25.20	3.06	5.53	0.08
59878	RES	SEG	m	50	3.92	0.39	0.21	0.15	0.31	3.16	13.64	54.99	3.38	0.93	0.89	50.50	8.28	0.20	0.19	24.20	1.60	2.73	0.00
59879	ADL	SEG	w	50	6.45	0.45	0.10	0.13	0.18	4.23	8.51	125.75	8.41	2.27	3.01	56.30	6.40	0.25	0.76	26.00	2.17	3.26	0.00
59880	RES	SEG	m	50	3.38	0.38	0.13	0.22	0.24	2.08	6.22	85.88	2.64	1.03	2.05	52.50	8.43	0.18	1.22	25.20	3.34	4.51	0.00
59881	ADL	SEG	m	50	4.64	0.34	0.13	0.23	0.18	2.44	14.56	77.53	4.51	1.49	1.75	51.70	3.33	0.25	0.31	25.60	2.98		0.00
59882	RES	SEG	w	50	4.74	0.33	0.15	0.10	0.21	3.96	8.45	95.16	12.25	4.86	5.15	56.60	11.36	0.39	0.85	23.50	2.18	5.66	0.00
59883	ADL	SEG	w	50	3.99	0.34	0.08	0.15	0.28	2.58	8.00	74.26	5.27	3.15	2.10	51.50	3.33	0.32	0.39	25.90	2.92	1.88	0.00
59885	ADL	SEG	w	50	6.29	0.34	0.27	0.08	0.24	2.83	12.49	101.04	1.56	1.09	1.56	54.60		0.37	0.25	25.90	3.12	3.63	0.00
59886	RES	SEG	m	50	3.33	0.45	0.22	0.11	0.32	3.77	9.69	73.87	2.82	1.46	2.43	54.20	6.99	0.24	0.68	25.10	1.64	8.62	0.00
59857	RES	HF	m	57	4.13	0.38	0.18	0.26	0.11	2.34	2.53	117.28	4.83	1.21	1.86	54.20	14.90	0.34	0.99	28.50	3.83	1.47	0.00
59858	RES	SEG	w	57	4.04	0.33	0.13	0.22	0.09	2.01	2.17	119.44	9.12	2.42	4.61	55.50	9.88	0.26	0.19	28.90	3.60	1.92	0.00
59859	RES	SEG	m	57	5.00	0.27	0.16	0.08	0.15	2.01	23.73	122.98	2.79	1.06	1.94	48.50	4.05	0.26	0.82	24.80	2.06	4.25	0.00
59860	RES	SEG	m	57	4.53	0.42	0.25	0.23	0.31	2.54	17.89	155.53	11.15	2.23	3.65	48.30	4.67	0.26	0.48	25.70	1.84	3.84	0.00
59861	RES	SEG	w	57	4.30	0.43	0.33	0.09	0.38	1.67	3.61	136.36	3.97	1.23	1.93	55.50	7.60	0.46	0.40	27.60	3.16	5.43	0.00
59862	ADL	SEG	m	57	5.07	0.48	0.17	0.14	0.29	3.02	18.19	182.46	4.80	0.94	2.14	48.40	6.21	0.32	0.51	26.00	2.62	3.92	0.00
59863	ADL	HF	w	57	4.35	0.32	0.27	0.07	0.31	3.71	19.10	221.86	6.60	0.82	0.62	52.00	2.88	0.27	0.76	27.00	2.12	2.55	0.00
59864	ADL	SEG	m	57	4.53	0.57	0.20	0.14	0.15	4.97	15.98	190.18	1.79	1.16	1.28	51.50	3.10	0.30	0.72	27.20	1.93	0.40	0.00
59866	ADL	SEG	w	57	4.67	0.45	0.27	0.13	0.37	3.46	26.05	121.45	15.42	1.93	4.60	50.20	7.94	0.42	0.97	25.10	2.44	2.83	0.00

Calf	Group	Breed	Sex	Day of life	Glucose (mmol/L)	Lactate (mmol/L)	BHB (mmol/L)	NEFA (mmol/L)	Triglycerides (mmol/L)	Cholesterol (mmol/L)	Insulin (uU/mL)	IGF-I (µg/L)	IGFBP3 (µg/L)	IGFBP2 (µg/L)	IGFBP4 (µg/L)	Total protein (g/L)	IgG1 (g/L)	IgG2 (g/L)	IgM (g/L)	Albumin (g/L)	Urea (mmol/L)	Fibrinoge (g/L)	Haptoglobin (g/L)
59867	ADL	SEG	m	57	4.56	0.35	0.14	0.17	0.26	2.46	11.84	166.60	2.71	1.13	1.65	48.90	8.28	0.25	0.69	26.80	3.10	0.00	0.00
59868	RES	SEG	m	57	3.81	0.84	0.01	0.30	0.24	2.15	12.29	79.35	1.51	1.10	1.40	50.00	9.88	0.30	0.37	26.40	3.56	4.08	0.00
59869	ADL	SEG	m	57	4.57	0.55	0.14	0.11	0.36	5.13	20.90	149.42	2.65	1.05	3.25	47.80	4.76	0.18	0.21	25.50	2.79	0.69	0.00
59870	RES	SEG	m	57	4.64	0.38	0.09	0.12	0.55	3.26	38.74	166.96	8.30	1.16	3.55	48.30	4.51	0.24	0.97	26.30	1.14	0.60	0.00
59871	ADL	HF	m	57	5.84	0.43	0.24	0.30	0.34	3.14	15.89	196.65	4.84	1.00	2.32	52.60	10.54	0.26	1.31	25.80	2.05	1.83	0.00
59872	RES	SEG	m	57	4.09	0.46	0.32	0.10	0.14	2.64	9.57	85.86	5.70	1.16	1.04	50.30	2.82	0.28	0.44	24.90	3.81	5.88	0.00
59873	ADL	SEG	m	57	4.70	0.47	0.16	0.13	0.24	3.10	19.80	151.95	12.02	2.01	2.00	50.90	6.21	0.21	0.36	25.30	2.77	7.84	0.00
59874	RES	SEG	m	57	3.31	0.38	0.07	0.11	0.24	3.28	4.79	62.77	1.64	1.33	1.59	49.80	10.18	0.38	1.00	23.40	2.90	4.09	0.00
59875	ADL	HF	m	57	5.04	0.94	0.17	0.17	0.21	3.07	10.40	111.80	2.06	0.97	1.09	52.20	11.56	0.37	0.56	26.00	1.92	2.00	0.00
59876	RES	HF	m	57	4.28	0.05	0.22	0.09	0.33	3.45	21.78	106.84	4.00	1.19	1.41	50.50	5.06	0.32	0.36	24.80	1.71	2.29	0.00
59877	ADL	SEG	w	57	4.37	0.33	0.22	0.02	0.19	2.05	16.72	113.82	3.51	1.46	2.04	53.80	7.16	0.39	0.68	26.00	3.73	2.94	0.00
59878	RES	SEG	m	57	4.37	0.53	0.27	0.11	0.30	3.04	19.19	70.00	3.92	1.21	0.10	51.60	9.55	0.19	0.29	25.10	2.83	2.22	0.00
59879	ADL	SEG	w	57	5.70	0.36	0.18	0.06	0.15	3.50	9.90	128.26	4.35	0.66	0.10	56.60	6.90	0.27	0.97	26.30	2.85	1.51	0.00
59880	RES	SEG	m	57	4.04	0.46	0.15	0.12	0.41	2.81	12.52	115.87	2.87	0.69	1.62	52.10	8.76	0.20	1.10	25.20	2.98	3.93	0.00
59881	ADL	SEG	m	57	4.02	0.43	0.10	0.10	0.19	1.49	11.29	122.42	3.77	1.09	1.46	52.00	4.12	0.25	0.43	25.90	3.58	4.00	0.00
59882	RES	SEG	w	57	5.13	0.37	0.16	0.09	0.23	3.72	12.07	116.18	11.63	5.04	5.73	55.50	11.67	0.44	1.07	24.10	2.03	1.22	0.00
59883	ADL	SEG	w	57	3.84	0.39	0.14	0.10	0.20	2.61	12.80	93.37	6.69	2.44	1.99	51.50	3.61	0.34	0.53	26.30	2.99	1.96	0.01
59885	ADL	SEG	w	57	7.40	0.53	0.17	0.18	0.31	2.10	47.97	95.52	1.85	1.14	1.38	52.50	11.56	0.38	0.35	26.50	4.00	2.00	0.00
59886	RES	SEG	m	57	2.94	0.43	0.18	0.11	0.29	3.50	4.83	73.54	2.86	1.04	2.06	53.70	6.99	0.40	0.95	24.40	1.49	6.25	0.00
