# Supplementary material for: Effects of Feeding Milk Replacer Ad Libitum or in Restricted Amounts for the First Five Weeks of Life on the Growth, Metabolic Adaptation, and Immune Status of Newborn Calves
Source: PLoS One. 2016 Dec 30;11(12):e0168974. doi: 10.1371/journal.pone.0168974 (PMC5201283; doi:10.1371/journal.pone.0168974)
Supplement: S4 Table — (PDF) [file pone.0168974.s005.pdf]

S4 Table. Complete data set of hepatic mRNA expression as shown in Table 4.

						Hepatic mRNA abundance given in arbitrary units														Concentration in liver (% wet weight)	
Calf	Group	Calf	Group	Breed	Sex	*G6PC*	*IGF1R*	*IGF1*	*PCCA*	*GHR*	*SLC2A2*	*IGFBP1*	*IGFBP2*	*IGFBP3*	*IGFBP4*	*INSR*	*PC*	*PCK2*	*PCK1*	Glykogen	Glucose
1	RES	59857	RES	HF	m	1.00	0.98	2.10	0.89	1.25	1.20	0.89	1.15	1.02	1.26	1.28	1.48	1.64	1.34	1.25	2.82
2	RES	59858	RES	SEG	w	1.06	0.77	1.30	1.01	1.10	0.92	0.84	1.06	0.85	1.27	0.99	0.90	1.35	0.86	1.28	1.36
3	RES	59859	RES	SEG	m	1.52	1.60	1.76	0.99	0.81	1.21	2.54	0.56	0.89	0.88	1.25	0.93	1.21	1.00	1.82	1.63
4	RES	59860	RES	SEG	m	1.00	1.13	1.43	1.53	0.82	0.73	1.00	0.90	1.09	1.06	1.10	0.81	1.14	1.65	4.51	0.17
5	RES	59861	RES	SEG	w	1.19	1.11	1.14	1.06	1.09	1.36	1.50	0.77	1.06	1.09	1.08	1.02	1.16	1.14	4.63	0.9
6	ADLIB	59862	ADL	SEG	m	0.81	0.92	1.19	1.17	1.27	0.88	1.87	0.99	0.93	1.13	0.96	1.16	0.93	1.50	3.43	1.96
7	ADLIB	59863	ADL	HF	w	0.75	0.86	1.04	0.95	1.01	1.03	3.14	1.01	0.91	1.15	0.93	0.91	0.82	1.10	2.87	1.79
8	ADLIB	59864	ADL	SEG	m	1.55	1.33	0.90	0.82	0.91	0.92	3.85	1.11	1.79	1.15	1.28	1.61	0.89	1.10	1.55	1.59
10	ADLIB	59866	ADL	SEG	w	1.49	1.01	1.06	1.00	1.24	1.07	3.84	1.04	1.11	0.99	1.07	1.18	1.32	2.50	2.96	1.36
11	ADLIB	59867	ADL	SEG	m	0.51	1.04	0.99	0.96	1.08	1.22	0.10	1.16	1.48	0.83	1.01	1.03	1.23	1.61	5.13	1.66
12	RES	59868	RES	SEG	m	1.97	1.19	1.05	0.93	0.55	1.55	1.26	1.14	0.62	1.00	1.08	1.11	0.77	0.57	5.94	1.29
13	ADLIB	59869	ADL	SEG	m	0.51	1.22	1.21	1.15	1.41	0.94	0.13	0.85	1.06	0.98	1.24	0.95	0.81	1.20	3.07	1.53
14	RES	59870	RES	SEG	m	0.51	0.98	1.82	0.77	1.28	0.81	0.22	0.44	1.14	0.89	1.04	0.88	0.69	1.10	4.57	1.19
15	ADLIB	59871	ADL	HF	m	1.40	1.09	1.53	1.23	1.70	1.20	3.74	0.92	1.22	1.05	1.14	1.15	1.12	1.07	6.51	1.39
16	RES	59872	RES	SEG	m	1.14	1.85	1.79	1.13	0.99	1.16	1.03	1.61	1.06	0.89	0.98	1.16	1.49	0.44	3.91	1.33
17	ADLIB	59873	ADL	SEG	m	1.38	1.06	1.16	0.90	0.73	1.39	1.15	1.37	1.08	0.99	0.94	1.03	1.01	1.57	3.10	1.13
18	RES	59874	RES	SEG	m	0.65	0.73	0.69	0.89	0.96	1.08	0.07	1.21	0.83	0.77	0.84	0.76	0.94	0.46	4.63	1.19
19	ADLIB	59875	ADL	HF	m	0.72	0.94	0.78	0.99	1.01	0.86	0.40	1.16	0.89	0.92	0.87	1.01	0.89	0.94	2.72	1.53
20	RES	59876	RES	HF	m	0.57	0.64	1.02	0.88	1.20	1.00	0.53	1.08	0.94	0.91	0.87	0.83	0.84	0.52	6.00	1.23
21	ADLIB	59877	ADL	SEG	w	0.82	0.73	0.93	1.18	0.78	0.77	0.27	0.93	0.86	1.08	1.08	1.00	1.09	0.77	1.70	1.53
22	RES	59878	RES	SEG	m	1.13	1.55	0.34	1.27	1.06	1.01	2.47	1.39	1.60	0.94	0.93	1.01	0.95	1.06	4.06	1.33
23	ADLIB	59879	ADL	SEG	w	0.52	1.64	0.92	1.10	0.99	0.88	0.89	0.68	0.82	0.87	1.31	0.63	0.87	1.07	3.13	1.53
24	RES	59880	RES	SEG	m	1.49	0.91	0.57	1.15	0.89	1.02	4.18	1.48	0.94	1.07	1.02	1.07	1.11	1.19	2.21	1.23
25	ADLIB	59881	ADL	SEG	m	0.78	0.93	0.44	0.91	0.61	0.75	1.30	0.98	0.73	1.03	0.93	1.07	1.07	0.75	2.03	1.16
26	RES	59882	RES	SEG	w	0.78	0.78	0.91	1.17	1.12	0.89	0.43	0.89	1.21	0.96	0.84	0.91	0.82	1.05	2.45	1.79
27	ADLIB	59883	ADL	SEG	w	2.07	0.73	0.74	0.94	0.80	0.92	2.43	1.23	0.83	1.01	0.91	1.24	0.97	1.14	0.90	1.46
29	ADLIB	59885	ADL	SEG	w	0.74	1.00	0.68	0.79	0.81	0.91	0.36	1.18	0.91	1.12	0.83	1.10	0.79	1.22	2.66	1.59
30	RES	59886	RES	SEG	m	0.75	1.10	0.86	0.96	0.94	1.14	2.98	1.12	0.88	1.01	0.83	1.06	0.88	0.46	3.76	1.03
